# Intense circulation of A/H5N1 and other avian influenza viruses in Cambodian live-bird markets with serological evidence of sub-clinical human infections

**DOI:** 10.1038/emi.2016.69

**Published:** 2016-07-20

**Authors:** Srey Viseth Horm, Arnaud Tarantola, Sareth Rith, Sowath Ly, Juliette Gambaretti, Veasna Duong, Phalla Y, San Sorn, Davun Holl, Lotfi Allal, Wantanee Kalpravidh, Philippe Dussart, Paul F Horwood, Philippe Buchy

**Affiliations:** 1 Institute Pasteur in Cambodia, Institute Pasteur International Network, Phnom Penh 12000, Cambodia; 2 National Veterinary Research Institute, Ministry of Agriculture, Forestry and Fisheries, Phnom Penh 12000, Cambodia; 3 Food and Agriculture Organization, Phnom Penh 12000, Cambodia; 4 Food and Agriculture Organization, Bangkok 10200, Thailand; 5 GlaxoSmithKline Pte Ltd, Singapore 189720, Singapore

**Keywords:** A/H5N1, A/H9N2, avian, Cambodia, influenza, live-bird markets, poultry, seroprevalence

## Abstract

Surveillance for avian influenza viruses (AIVs) in poultry and environmental samples was conducted in four live-bird markets in Cambodia from January through November 2013. Through real-time RT-PCR testing, AIVs were detected in 45% of 1048 samples collected throughout the year. Detection rates ranged from 32% and 18% in duck and chicken swabs, respectively, to 75% in carcass wash water samples. Influenza A/H5N1 virus was detected in 79% of samples positive for influenza A virus and 35% of all samples collected. Sequence analysis of full-length haemagglutinin (HA) and neuraminidase (NA) genes from A/H5N1 viruses, and full-genome analysis of six representative isolates, revealed that the clade 1.1.2 reassortant virus associated with Cambodian human cases during 2013 was the only A/H5N1 virus detected during the year. However, multiplex reverse transcriptase-polymerase chain reaction (RT-PCR) analysis of HA and NA genes revealed co-circulation of at least nine low pathogenic AIVs from HA1, HA2, HA3, HA4, HA6, HA7, HA9, HA10 and HA11 subtypes. Four repeated serological surveys were conducted throughout the year in a cohort of 125 poultry workers. Serological testing found an overall prevalence of 4.5% and 1.8% for antibodies to A/H5N1 and A/H9N2, respectively. Seroconversion rates of 3.7 and 0.9 cases per 1000 person-months participation were detected for A/H5N1 and A/H9N2, respectively. Peak AIV circulation was associated with the Lunar New Year festival. Knowledge of periods of increased circulation of avian influenza in markets should inform intervention measures such as market cleaning and closures to reduce risk of human infections and emergence of novel AIVs.

## INTRODUCTION

Avian influenza viruses (AIVs) naturally infect the gastrointestinal tracts of wild birds from the Orders Anseriformes (ducks, geese and swans) and Charadriformes (waders and gulls). Within these hosts, 16 haemagglutinin (HA) and nine neuraminidase (NA) surface glycoprotein types have been described. The HA and NA types carried by the virus are used for classification and can be found in various combinations, such as A/H5N1 and A/H3N2. Wild birds have been implicated in the global dissemination of AIVs and, in some instances, the introduction of AIVs into domestic poultry populations.^1^ Although low pathogenic avian influenza (LPAI) viruses (which have limited impact on poultry mortalities) constitute the majority of these viruses, highly pathogenic avian influenza (HPAI) viruses such as A/H5N1 can result in mortalities approaching 100% when introduced into domestic flocks.^2^

Influenza A viruses, which constitute all of the known AIVs, have a segmented genome with eight separate RNA strands enclosed in the virus. When an animal is co-infected with two different influenza viruses, reassortment of these RNA segments can occur, resulting in a new virus with characteristics distinct from the parent viruses.^3^ The 2009 emergence and resulting pandemic from A/H1N1pdm09 virus was caused by a virus that underwent multiple reassortment events with strains from pigs, birds and humans before suddenly gaining the ability to efficiently transmit between humans.^4^ Pandemic events resulting from reassortment of influenza viruses have occurred at least three times in the last 100 years,^5, 6^ resulting in high human morbidity and mortality worldwide. The most severe influenza pandemic in recorded history, the 1918 ‘Spanish flu', is thought to have arose from an avian influenza strain that directly adapted to the human host.^7^ Clearly it is imperative that close monitoring of reassortments and mammalian-adapted mutations in avian influenza strains is needed.

Since the emergence of HPAI A/H5N1 in Southern China in 1996 and 1997,^8^ descendants of this virus have caused considerable economic losses in poultry populations, primarily in east and southeast Asia, but also in Africa and Europe. Influenza A/H5N1 has been identified as a significant threat for pandemic emergence. To date, at least 844 people have been infected worldwide, resulting in 449 deaths (53% case fatality rate). The virus was first detected in Cambodian poultry in early 2004 and the first human cases were detected in 2005.^9^ As of December 2015, 56 human cases (including 37 deaths) and 43 poultry outbreaks of influenza A/H5N1 have been recorded in the country.^10, 11^

Live-bird markets (LBMs) have been implicated as an important source of human sporadic cases and dissemination of avian influenza since the emergence of influenza A/H5N1.^12^ Previous studies have established that LBMs serve as hubs for the circulation and persistence of AIVs through the presence of multiple avian species, the constant introduction of immunologically naive hosts and the frequent lack of biosecurity measures.^1, 13, 14^ Recent surveillance studies have documented that A/H5N1 and A/H7N9 commonly co-circulate with other subtypes of avian influenza in LBMs,^15, 16, 17^ thus increasing the likelihood of reassortment events.

In the present study we initiated poultry and environmental surveillance for A/H5N1 and other AIVs in four LBMs in Cambodia to better ascertain virus circulation in this setting. A longitudinal human serosurvey was also conducted to investigate the risk of human avian influenza infections in exposed LBM workers.

## MATERIALS AND METHODS

### Animal and environmental samples

**Figure 1:**
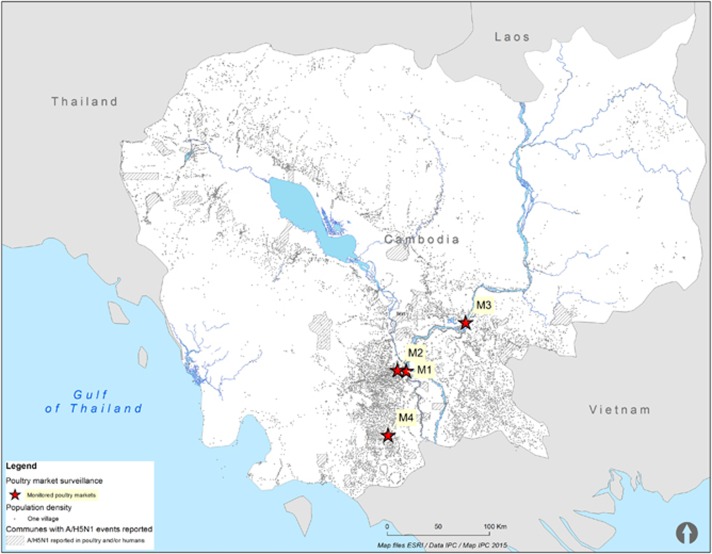
Map showing communes with reported confirmed human A/H5N1 cases during 2006–2014, population density (indicated by the number of villages) and live-bird markets investigated in 2013.

**Figure 2:**
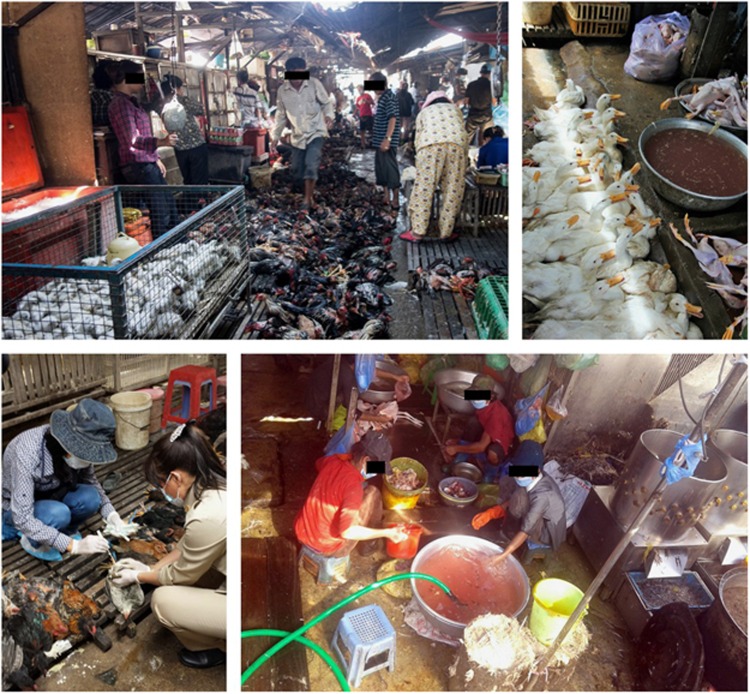
Live-bird markets in Cambodia typically have poor biosecurity, with multiple animal species slaughtered onsite. Animal and environmental sampling in these markets is crucial to monitor virus circulation and the emergence/introduction of new viruses.

Commencing in January 2013, through November 2013, environmental and poultry samples were collected at four LBMs in Cambodia: an LBM in central Phnom Penh (M1), a wholesaling farm/slaughter house in Phnom Penh (M2), an LBM in Kampong Cham province (M3) and an LBM in Takeo province (M4) (Figure 1). These LBMs were selected for the study as they represent the largest poultry collection sites in the most densely populated region of the country. Samples were collected from each market every 1–2 weeks (Figure 1). The LBMs in Cambodia typically have poor biosecurity, with slaughtering of animals onsite occurring for a range of domestic animals (Figure 2). Birds are typically sourced from backyard flocks by middlemen who transport the animals through a convoluted system of semi-commercial farms and stock houses before being transported to the main LBMs.^18^ Thus, tracking the original source of poultry is usually not possible.

During each market investigation, tracheal and cloacal swabs were collected (both samples pooled in one tube of viral transport medium for each animal) from four randomly selected poultry (three ducks and one chicken). Environmental samples were also collected in the same cage/site where the poultry swabs were collected to investigate contamination of the LBMs with AIVs. During each mission, 50 ml of carcass wash water (water used to wash the carcasses once the poultry has been slaughtered and defeathered), 50 ml of poultry drinking water (small bowl of water placed in cages), soil/mud from an area within 50cm around the poultry cages/poultry resting area and samples of discarded feathers were collected. The water, soil/mud and feather samples were processed and nucleic acids extracted following techniques described previously.^19, 20^ Extracts were then tested by quantitative real-time reverse-transcriptase polymerase chain reaction (qRT-PCR) for the detection of M, H5 and N1 genes.

M-gene-positive samples, for which there was sufficient sample and high virus concentration (CT<30; *n*=78), were inoculated into specific pathogen-free embryonated chicken eggs for virus isolation.^21^ Isolates were then tested using influenza universal multiplex RT-PCR assays to test for all known subtypes of influenza.^22^ Universal multiplex RT-PCR typing assays were also applied to the original samples where isolation could not be achieved but discordance between M-gene and H5 qRT-PCR testing suggested the presence of a non-H5 AIV. The amplified RT-PCR products from isolates and original material were submitted to a commercial sequencing facility (Macrogen, Seoul, South Korea) for sequencing by the Sanger method. Full-genome sequences were generated from representative A/H5N1 isolates using methods previously described.^9, 23^

Contiguous sequences were assembled using CLC Workbench (CLC bio) and compared to representative influenza virus sequences downloaded from the NCBI GenBank database. Neighbour-joining trees were constructed with MEGA5^24^ and bootstrap values were calculated and expressed as a percentage from 1000 replicates.

### Human samples

The longitudinal human serological study was approved by the National Ethics Committee for Human Research (approval NO. 267, 24 December 2012). Serum samples were collected, after obtaining informed consent, from LBM workers at the start of the study (January 2013) to form a baseline, and 8 weeks after the three major national festivals shown by previous work to be associated with increased A/H5N1 circulation in markets:^25^ Lunar New Year, week 6; Khmer New Year, week 15; Pchum Ben, week 40. All adult-age LBM sellers or workers were exhaustively recruited in the four targeted live-poultry markets. The sample size could not be calculated as transmission to humans was unlikely and its probability in Cambodia is unknown. Participants were informed to report any acute febrile, respiratory or digestive signs, and were provided with a toll-free phone number.

Serum samples were tested for avian influenza A/H5N1, A/H9N2 and A/H7N9 antibodies using the haemagglutination inhibition assay (HIA) and microneutralization assay (MN). The HIA and MN testing were performed using influenza A/H5N1 clade 1.1.2 reassortant viruses (A/Cambodia/X0121311/2013 and A/Cambodia/ X0125302/2013), which were isolated from human cases during 2013, and influenza A/H9N2 virus (A/Environment/Cambodia/E265/2013), which was isolated from the LBMs during 2013. The HIA and MN testing for A/H7N9 were performed using the strain A/Anhui/01/2013, supplied through the World Health Organization (WHO) Global Influenza Surveillance and Response System (Dr Sylvie van der Werf, Department of Virology, Institut Pasteur, Paris, France). Exposure to these viruses was considered ‘confirmed' with a HIA titre of ≥80 and a MN titre of ≥40. A seroconversion was defined as the detection of antibodies equal to or above the thresholds defined above following no detection of antibodies in the serum sample from the previous period.

Laboratory data were entered using an Excel spreadsheet (Microsoft Excel, Microsoft, Redmond, WA, USA). A baseline assessment was documented using point prevalence for influenza antibodies, and incidence rates during follow-up were then computed using data on laboratory-confirmed seroconversions in LBM workers (numerator), and the number of days elapsed during the last serosurvey (denominator). Poisson confidence intervals for the incidence rates were computed using Stata 11 (Stata Corp., College Station, TX, USA) with the function cii for the binomial CI, and the functions cii and the option ‘poisson' for the poisson CI.

## RESULTS

### Animal and environmental surveillance of A/H5N1

**Figure 3:**
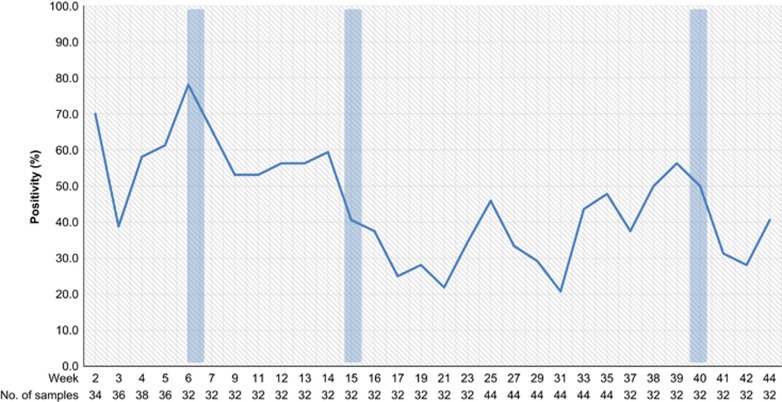
Proportion of samples positive for Influenza A virus (M-gene) for each sampling mission at four live-bird markets in Cambodia, 2013. The major Cambodian festivals are during week 6 (Lunar New Year festival), week 15 (Khmer New Year festival) and week 40 (Pchum Ben festival), and are indicated by vertical blue bars.

During the study, a total of 1048 samples were collected, with 45% of all samples positive for influenza A RNA by qRT-PCR (Table 1). Influenza A viruses were detected in at least one sample during 93% of 120 collection missions, with influenza virus detected in three of the markets (M1, M2 and M3) on all but one sampling mission and detected in 83% of sampling missions from the remaining market (M4). Influenza A (M-gene) ribonucleic acid (RNA) was detected most frequently in carcass wash water samples (75%, *n*=146), followed by feathers (61%, *n*=138), poultry drinking water (50%, *n*=138), soil/mud (48%, *n*=146), duck swabs (32%, *n*=358) and chicken swabs (18%, *n*=122). H5 and N1 genes were detected in 79% (*n*=372) and 58% (*n*=270), respectively, of the 468 samples that tested positive for influenza A (M-gene), which accounted for 35% and 26% of all samples collected, respectively. Peak AIV circulation was detected from January through March, with particularly high detection rates during the Lunar New Year festival period (Figure 3).

Influenza A/H5N1 virus was isolated from 71% (55/78) of samples from which H5 was detected and isolation was attempted. Isolation was only attempted on high viral load samples (M-gene qRT-PCR CT value <30) and success rates differed considerably by sample type, with isolation being highest in duck swabs (83%, 19/23), followed by poultry drinking water (80%, 8/10), soil/mud (72%, 13/18), feathers (64%, 7/11), carcass wash water (53%, 8/15) and chicken swabs (0%, 0/1).

**Figure 4:**
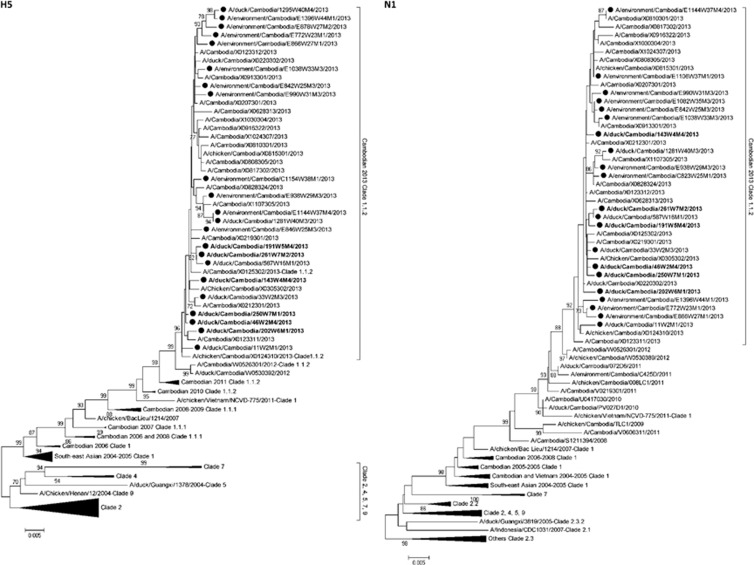
Neighbour-joining phylogenetic tree of the HA and NA genes of highly pathogenic avian influenza A/H5N1 viruses detected during live-bird market surveillance in Cambodia. Viruses collected during the present market study are denoted by a black circle. Viruses for which the full genome is available are denoted in bold. Mid-point rooted phylogenetic trees were constructed in MEGA5. Bootstraps >70 generated from 1000 replicates are shown at branch nodes. The scale bar represents the number of nucleotide substitutions per site.

Full-gene sequences were generated for H5 (*n*=22) and N1 (*n*=21) for all viruses detected in the study where sufficient virus concentration allowed for successful sequencing (Figure 4).^10, 23^ Full-genome sequences (eight fragments) were generated for six influenza A/H5N1 viruses detected during the study (Supplementary Figure S1). For other viruses full-gene sequences could not be generated for all fragments (Supplementary Table S1). All A/H5N1 viruses detected in the study clustered with the clade 1.1.2 reassortant strains associated with human cases and poultry outbreaks of A/H5N1 during 2013.^10^

### Non-H5 avian influenza detection

**Figure 5:**
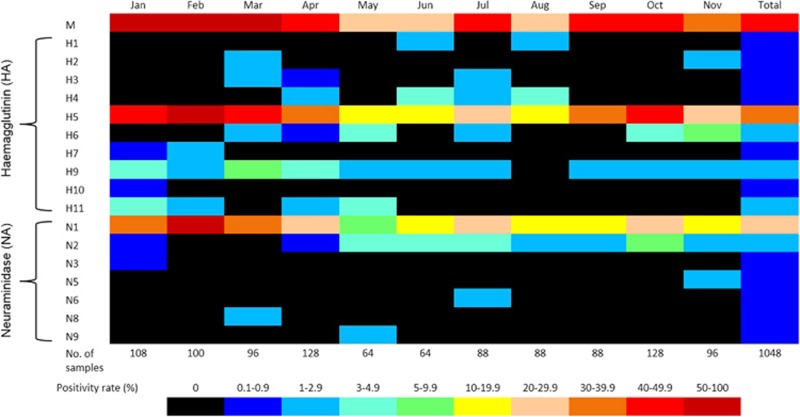
Heat map of the avian influenza virus positivity rate in four Cambodian live-poultry markets, 2013.

A large number and variety of non-H5 avian influenza strains were detected during the study. Full-HA sequences were generated from two H1, two H2, three H3, nine H4, 15 H6, one H7, 27 H9, one H10 and twelve H11 viruses (Supplementary Figure S2). Full NA sequences from twenty-two N2, one N3, one N5, two N6, one N8 and two N9 subtypes were also generated from the same samples (Supplementary Figure S3). However, as many of the environmental samples contained evidence of multiple avian influenza strains, it was difficult to ascertain that HA and NA sequences derived from the same sample actually belonged to the same virus strain. Co-infections between AIVs were not detected in any poultry samples. Many other partial HA and NA sequences were also generated, but not included in these results due to difficulties in determining close phylogenetic relationships from incomplete gene sequences. AIVs were detected throughout the year (Figure 5), with a distinct peak of activity during January−March, perhaps mostly due to the increased circulation of A/H5N1 during major Cambodian festivals, as previously reported.^25^

### Longitudinal human serosurvey

Successive serological surveys in the poultry worker cohort provided evidence of seroconversions and some prior exposure (Table 2). At baseline sampling (January 2013), 125 participants were enrolled in the study, with one person testing positive to A/H5N1 antibodies and another testing positive to A/H9N2 antibodies. Participant retention was high throughout the study, with 117, 105 and 106 people resampled at the second (March 2013), third (June 2013) and fourth (November 2013) sampling missions, respectively. Seroconversions to A/H5N1 were detected for two participants at the third sampling and two participants at the fourth sampling. Seroconversions to A/H9N2 were detected for one participant at the third sampling. Overall seroprevalence was 4.5% for A/H5N1 and 1.8% for A/H9N2. Rates of seroconversion were 3.7 infections per 1000 person-months for A/H5N1 and 0.9 infections per 1000 person-months for A/H9N2. There was no serological evidence of exposure or molecular detection of A/H7N9 before or during the study.

There were no calls to the toll-free number reporting an incident with clinical signs or symptoms compatible with influenza infection.

## DISCUSSION

LBMs have been established as important foci for the transmission of AIVs and the potential emergence of reassortant strains.^13^ The presence of multiple host species and the continued introduction of naive birds create an ideal environment for the persistence and emergence of AIVs. Exposure to live poultry has been associated with fatal human A/H5N1 infections, which for instance led to the government of Hong Kong rapidly closing LBMs in 1997 and slaughtering large numbers of poultry.^26^ The LBMs surrounding Phnom Penh have been established as foci for poultry movement in the country^18, 27^ and a previous market surveillance study in 2011 revealed a high rate of influenza A/H5N1 circulation in this setting.^25^

To our knowledge, AIVs were detected in Cambodian LBMs during 2013 at a higher frequency than any other study published previously,^25, 28, 29, 30, 31, 32, 33, 34^ with 45% of all samples (poultry swab and environmental samples), 32% of individual duck swabs and 18% of chicken swabs positive for influenza A RNA. The majority of the AIVs detected were likely A/H5N1, with 35% of samples positive for H5 qRT-PCR. Although there was clearly high co-circulation of LPAI viruses, much of the difference between the detection rates of M-gene, H5 and N1 was probably due to the differing qRT-PCR assay sensitivities (M>H5>N1). We previously detected high circulation of A/H5N1 in these same markets in 2011.^25^ However, in 2013 there was a >2.5-fold increase in the frequency with which the virus was detected despite the same sample processing, nucleic acid extraction and RT-qPCR methods being used.

In 2013, Cambodia had the highest confirmed human A/H5N1 caseload per capita in the World. During 2013 alone, 26 human A/H5N1 cases (14 deaths) were detected in Cambodia, a dramatic increase in the 21 total cases that had been detected in the preceding 8 years, 2005–2012. This rise in reported human cases of A/H5N1 coincided with the emergence of a reassortant virus that contained the HA and NA genes from the previously circulating clade 1.1.2 genotype Z virus, and the matrix and internal genes from a clade 2.3.2.1 virus previously circulating in southern Vietnam.^10^ Sequence and phylogenetic analyses of the HA and NA genes (Figure 4) and the matrix and internal genes (Supplementary Figure S1) from the A/H5N1 market strains revealed that all of the viruses clustered closely with other clade 1.1.2 reassortant strains associated with poultry outbreaks and human cases in 2013.^10^ Questions remain regarding the causes of the dramatic increase in human cases in 2013 and whether the reassortant strain is more transmissible in poultry, resulting in the intense circulation that we observed in this report. Alternatively, increased surveillance and education of clinicians may have resulted in improved detection of human A/H5N1 cases. Presently we do not know the exact role played by LBMs in the infection of poultry workers and market clients.

Similarly to our previous LBM surveillance in 2011,^25^ increased circulation of AIVs was detected before and during the major Cambodian festivals (Figure 3). In particular, increased circulation of influenza A viruses was detected during the period between the Lunar New Year and Khmer New Year festivals. A previous study on the poultry trade links in Cambodia established that there was a significant increase in the trade volume of poultry prior to these festivals, with an assumed rise in cross-border poultry trade in response to increased demand.^18^ The greater volume of poultry trade and the expansion of poultry trading networks during these festival periods present opportunities for virus spread throughout domestic flocks and the introduction of new strains of AIVs. Peaks in AIV levels were also observed during times that were not associated with known festivals (e.g. week 25 and 35). Climatic factors, which were not analysed in this study, are also likely to have an influence on levels of AIV circulation. Knowledge of these periods of intense circulation should inform future control policies such as targeted poultry vaccination, quarantining and improved market cleaning. Such measures have proven effective in reducing avian influenza viral isolation rates in LBMs^35, 36, 37^ and may reduce the spread of AIVs back to farms through fomites and personnel.^38^

Environmental sample testing showed that the LBM environment is highly contaminated with AIVs (Table 1). Influenza A detection rates were highest in carcass wash water samples, which serve as ‘pooled' samples for multiple slaughtered birds. During the slaughtering process at the markets, birds are defeathered and eviscerated before carcasses are washed in a large bucket of water. We observed that 20–30 carcasses were often washed in the same container of water before it was refreshed. These samples were positive for influenza A RNA in 75% of samples from all four markets (ranging from 63 to 86%). Poultry drinking water was also a useful surveillance sample, with 50% of samples testing positive for influenza A RNA; A/H5N1 isolation rates were higher when compared to carcass wash water. Detection rates and isolation rates were also high with soil/mud and discarded feather samples, but these specimens required more complex processing before testing. High contamination of the LBM environment, including relatively high rates of virus isolation, is evidence that the risk of human exposure is very high. Carcass wash water and poultry drinking water samples proved a useful adjunct to poultry swabs for monitoring purposes in the LBMs.

Based solely on the HA typing, analysis of LBM samples collected in 2013 revealed the presence of at least nine other subtypes of AIVs co-circulating with A/H5N1 (Figure 5). This situation increases the likelihood of reassortment events occurring, which may result in the emergence of new influenza subtypes. The recent emergence of reassortant HPAI subtypes of H5, such as A/H5N2, A/H5N5, A/H5N6 and A/H5N8,^39, 40, 41, 42, 43^ is evidence of the risk of new viruses arising through reassortment events with A/H5N1. The sudden emergence of multiple H5 subtypes has not been adequately explained but could be related to the diversity of AIVs currently circulating in domestic poultry populations. The emergence of A/H7N9 virus in China in early 2013^44^ has also prompted increased concerns about the possibility of a pandemic virus emerging in the Asia-Pacific region. Although most of the non-H5 AIVs detected in this study likely pose little or no risk to humans, the high prevalence of multiple avian influenza strains is an indictment on the poor biosecurity associated with poultry rearing and selling in the region. Previous studies and surveillance have also established that there is intense circulation of poultry between regions and across borders, which facilitates the regional spread of AIVs.^9, 23^

In our study, the detection of A/H5N1 was achieved using qRT-PCR, whereas the detection of other AIVs was done using universal multiplex RT-PCR assays with comparatively reduced sensitivity. In addition, multiplex testing was only conducted where there was disparity between M-gene and H5 qRT-PCR assays. Furthermore, due to the difficulties with classifying influenza viruses using incomplete gene sequences we have only reported LPAI detection where we have been able to generate full HA or NA sequences. It is therefore likely that the true prevalence of non-H5 AIVs was far greater than what we have reported in these market samples. The potential for reassortment between AIVs, including A/H5N1, could result in the emergence of viral strains with considerable impacts on poultry and/or human health. This study, coupled with other recent studies in LBMs from other Asian countries,^15, 16, 17^ confirms that the environment of LBMs provides a pool of genes for potential emergence of new pandemic viruses. The silent circulation of a multitude of LPAI viruses, which remain undetected and unmonitored in most Asian countries, heightens the threat when they co-circulate with pandemic candidates such as A/H5N1 and A/H7N9.

As no poultry workers reported any symptoms in relation to acute avian influenza infection, seroconversions in this study would most likely be related to sub-clinical or very mild cases. It has been reported that asymptomatic and mild avian influenza infections lead to seroconversions with low antibody titres that quickly decrease below the threshold recommended by WHO for a confirmed A/H5N1 case (HIA≥160; MN≥80).^45^ For this reason, we considered as positive all individuals with a HIA titre of ≥80 and a MN titre of ≥40, which is consistent^46^ or more stringent^47, 48, 49, 50, 51, 52^ than the cut-off levels suggested by other authors. However, it is difficult to directly compare the results between studies as there is no consensus on the antibody titre that results from a mild or asymptomatic infection, and consistent cut-off levels have not been used in past studies. According to our classification, 4.5% of workers who participated in the study had antibodies against A/H5N1. This prevalence is much higher than that reported among LBM workers in Egypt,^53^ Thailand^47^ or China,^54^ and much higher than in villagers in Thailand^51^ or Cambodia.^48^ The incidence was 3.7 per 1000 person-months in our study, a figure twice that described in Bangladesh in 2009–2010.^46^ There were no reported clinical signs in our cohort, confirming that a large proportion of human A/H5N1 infections may go undetected. Poultry workers in Cambodia and other Asian countries, where there is endemic circulation of A/H5N1, are constantly exposed to high levels of virus. Although this may lead to mild or sub-clinical infections with seroconversions, it seems that transmission to humans resulting in acute infections is still rare.

Influenza A/H9 viruses, predicted to be A/H9N2 based on the phylogenetic analysis of the HA and NA sequences repeatedly detected in the same samples, were detected in 2.6% of samples analysed in this study. Although A/H9N2 viruses have low pathogenicity for poultry, novel HPAI and LPAI viruses affecting humans, such as A/H5N1, A/H7N9 and A/H10N8, contain internal genes originating from influenza A/H9N2.^55, 56, 57^ This may have facilitated the ability of these viruses to cause infection and deaths in humans. Human infections with A/H9N2 have been reported in the literature, and seroprevalence studies have suggested that asymptomatic or mild infections commonly occur in high-risk locations such as LBMs and slaughterhouses.^50, 53, 58, 59^ The detection of A/H9N2 antibodies in 1.8% of LBM workers participating in our study and an incidence of 0.9 infections per 1000 person-months confirm other data on poultry-to-human transmission of A/H9N2,^53, 59^ including among rural villagers in Cambodia.^48^ In addition, A/H9N2 infections have also been detected in other mammals such as guinea pigs, dogs, horses and pigs.^59, 60^ The ability of A/H9N2 to cross the species barrier and the relatively high frequency in which it has been implicated in reassortment events suggest that this virus significantly contributes to the emergence of viruses that pose an important public health risk and should therefore be very closely monitored.

In this study we document intense co-circulation of influenza A/H5N1 and LPAI viruses in Cambodian LBMs during 2013. In addition, serological surveys provided evidence of sub-clinical A/H5N1 and A/H9N2 infections. Interventions such as regular cleaning/disinfection, bans on overnight poultry storage, targeted closure during periods of peak circulation and segregation of poultry slaughtering areas should be considered in LBMs to reduce the threat of the emergence of AIVs with public health or animal health impacts. Further monitoring of the circulation of influenza A/H5N1 in Cambodian LBMs and research into the mechanisms associated with human cases is warranted.

## Figures and Tables

**Table 1:** Results of qRT-PCR tests on environmental samples and poultry swab specimens collected from four live poultry markets in Cambodia, 2013 (results displayed by sample type and by market)

**Sample type**	**Market ID**^a^	**Number of samples positive: M (*****%***)	**Number of samples positive: H5 (*****%***)	**Number of samples positive: N1 (*****%***)	**Isolation rate of A/H5N1 from qRT-PCR high viral load samples**^b^
Poultry drinking water	M1	21/36 (58)	18/36 (50)	11/36 (31)	80% (8/10)
	M2	14/36 (39)	9/36 (25)	8/36 (22)	
	M3	19/36 (53)	14/36 (39)	10/36 (28)	
	M4	15/30 (50)	14/30 (47)	9/30 (30)	
	All 4 M	69/138 (50)	55/138 (40)	38/138 (28)	
					
Carcass washing water	M1	32/39 (82)	31/39 (79)	27/39 (69)	53% (8/15)
	M2	25/40 (63)	19/40 (48)	9/40 (23)	
	M3	31/36 (86)	29/36 (81)	26/36 (72)	
	M4	22/31 (71)	22/31 (71)	17/31 (55)	
	All 4 M	110/146 (75)	101/146 (69)	79/146 (54)	
					
Soil/mud	M1	22/39 (56)	18/39 (46)	16/39 (41)	72% (13/18)
	M2	26/40 (65)	18/40 (45)	13/40 (33)	
	M3	13/36 (36)	10/36 (28)	8/22 (22)	
	M4	9/31 (29)	9/31 (29)	5/31 (16)	
	All 4 M	70/146 (48)	55/146 (38)	42/146 (29)	
					
Feathers	M1	27/36 (75)	23/36 (64)	17/36 (47)	64% (7/11)
	M2	24/36 (67)	19/36 (53)	11/36 (31)	
	M3	21/36 (58)	19/36 (53)	10/36 (28)	
	M4	12/30 (40)	11/30 (37)	7/30 (23)	
	All 4 M	84/138 (61)	72/138 (52)	45/138 (33)	
					
Duck swab	M1	34/90 (38)	30/90 (33)	22/90 (24)	83% (19/23)
	M2	26/97 (27)	10/97 (10)	8/97 (8)	
	M3	30/90 (33)	17/90 (19)	15/90 (17)	
	M4	23/81 (28)	20/81 (25)	13/81 (16)	
	All 4 M	113/358 (32)	77/358 (22)	58/358 (16)	
					
Chicken swab	M1	8/30 (27)	6/30 (20)	5/30 (17)	0% (0/1)
	M2	4/23 (17)	2/23 (9)	1/23 (4)	
	M3	8/30 (27)	3/30 (10)	1/30 (3)	
	M4	2/39 (5)	1/39 (3)	1/39 (3)	
	All 4 M	22/122 (18)	12/122 (10)	8/122 (7)	
					
Total	M1	144/270 (53)	126/270 (47)	98/270 (36)	71% (55/78)
	M2	119/272 (44)	77/272 (28)	50/272 (18)	
	M3	122/264 (46)	92/264 (35)	70/264 (27)	
	M4	83/242 (34)	77/242 (32)	52/242 (21)	
	All 4 M	468/1048 (45)	372/1048 (35)	270/1048 (26)	

Abbreviation: quantitative reverse transcriptase-polymerase chain reaction, qRT-PCR.

a Market identification (ID): M1, an LBM in central Phnom Penh; M2, a wholesaling farm/slaughter house in Phnom Penh; M3, an LBM in Kampong Cham province; M4, an LBM in Takeo province; 4 M, pooled results of these four markets.

b Samples were inoculated into embryonated chicken eggs for virus isolation when the M-gene qRT-PCR CT value was <30.

**Table 2:** Positive test results in participants and estimated seroprevalence and incidence rate, Cambodian live-bird markets study, 2013

**Viruses tested**^a^	**Number positive**^b^	**Number of people sampled**^c^	**Global percentage of positives**	**Binomial 95% CI**	**Number of seroconversions**	**Participation (person-month)**^d^	**Incidence (cases per 1000 person-months)**	**Poisson 95% CI**
A/H5N1	5	111	4.50	1.5–10.2	4	1079	3.71	1.0–9.5
A/H9N2	2	111	1.80	0.2–6.4	1	1083	0.92	0.0–5.1
A/H7N9	0	111	0.00	0.0–3.3^e^	0	1102	0.00	0.0–3.3^e^
Total	7	111	6.31	2.6–12.6	5	1063	4.70	1.5–11.0

Abbreviation: confidence interval, CI.

a A/H5N1 Clade 1.1.2 reassortant strains, A/Cambodia/X0121311/2013 and A/Cambodia/X0125302/2013; A/H9N2 strain, A/Environment/Cambodia/E265/2013; A/H7N9 strain, A/Anhui/01/2013.

b Exposure to these viruses was considered positive with a haemagglutination inhibition assay titre of ≥80 and a microneutralization assay titre of ≥40.

c Only participants who provided samples for at least two time points were included in these analyses.

d Participants were removed from further calculations once they were recorded as ‘positive' in the assumption that antibodies are protective against further infections.

e One-sided, 97.5% CI.
