# Supplementary material for: Intense circulation of A/H5N1 and other avian influenza viruses in Cambodian live-bird markets with serological evidence of sub-clinical human infections
Source: Emerg Microbes Infect. 2016 Jul 20;5(7):e70–. doi: 10.1038/emi.2016.69 (PMC5141262; doi:10.1038/emi.2016.69)
Supplement: Supplementary Figure S1 [file emi201669x1.pdf]

PB2

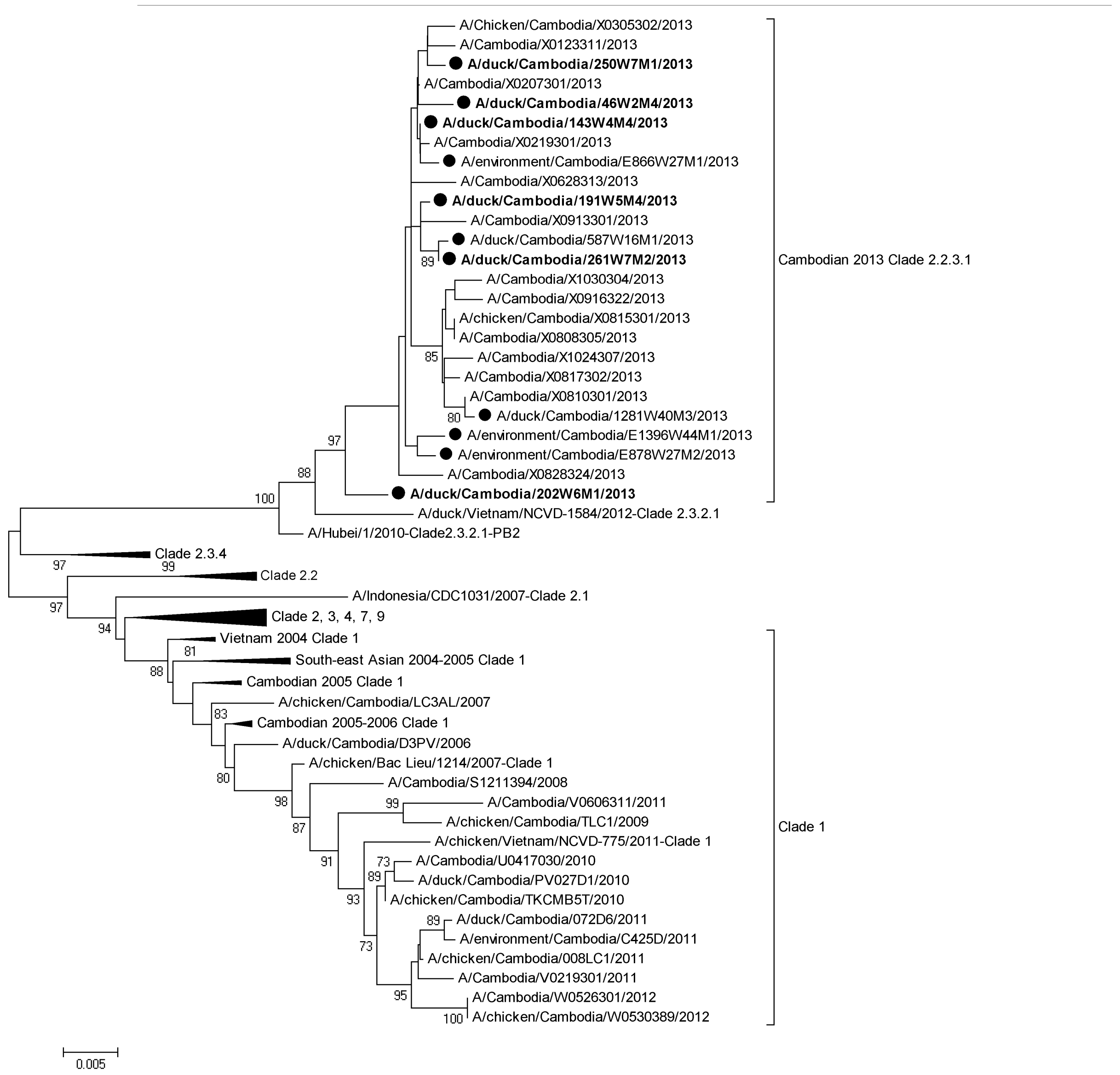
A/Chicken/Cambodia/X0305302/2013
A/Cambodia/X0123311/2013
A/duck/Cambodia/250W7M1/2013
A/Cambodia/X0207301/2013
A/duck/Cambodia/46W2M4/2013
A/duck/Cambodia/143W4M4/2013
A/Cambodia/X0219301/2013
A/environment/Cambodia/E866W27M1/2013
A/Cambodia/X0628313/2013
A/duck/Cambodia/191W5M4/2013
A/Cambodia/X0913301/2013
A/duck/Cambodia/587W16M1/2013
A/duck/Cambodia/261W7M2/2013
A/Cambodia/X1030304/2013
A/Cambodia/X0916322/2013
A/chicken/Cambodia/X0815301/2013
A/Cambodia/X0808305/2013
A/Cambodia/X1024307/2013
A/Cambodia/X0817302/2013
A/Cambodia/X0810301/2013
A/duck/Cambodia/1281W40M3/2013
A/environment/Cambodia/E1396W44M1/2013
A/environment/Cambodia/E878W27M2/2013
A/Cambodia/X0828324/2013
A/duck/Cambodia/202W6M1/2013
A/duck/Vietnam/NCVD-1584/2012-Clade 2.3.2.1
A/Hubei/1/2010-Clade2.3.2.1-PB2
Cambodian 2013 Clade 2.2.3.1
Clade 2.3.4
Clade 2.2
A/Indonesia/CDC1031/2007-Clade 2.1
Clade 2, 3, 4, 7, 9
Vietnam 2004 Clade 1
South-east Asian 2004-2005 Clade 1
Cambodian 2005 Clade 1
A/chicken/Cambodia/LC3AL/2007
Cambodian 2005-2006 Clade 1
A/duck/Cambodia/D3PV/2006
A/chicken/Bac Lieu/1214/2007-Clade 1
A/Cambodia/S1211394/2008
A/Cambodia/V0606311/2011
A/chicken/Cambodia/TLC1/2009
A/chicken/Vietnam/NCVD-775/2011-Clade 1
A/Cambodia/U0417030/2010
A/duck/Cambodia/PV027D1/2010
A/chicken/Cambodia/TKCMB5T/2010
A/duck/Cambodia/072D6/2011
A/environment/Cambodia/C425D/2011
A/chicken/Cambodia/008LC1/2011
A/Cambodia/V0219301/2011
A/Cambodia/W0526301/2012
A/chicken/Cambodia/W0530389/2012
Clade 1
89
85
80
97
88
100
97
99
97
94
81
88
83
80
98
87
91
99
73
89
93
73
89
95
100
0.005

PB1

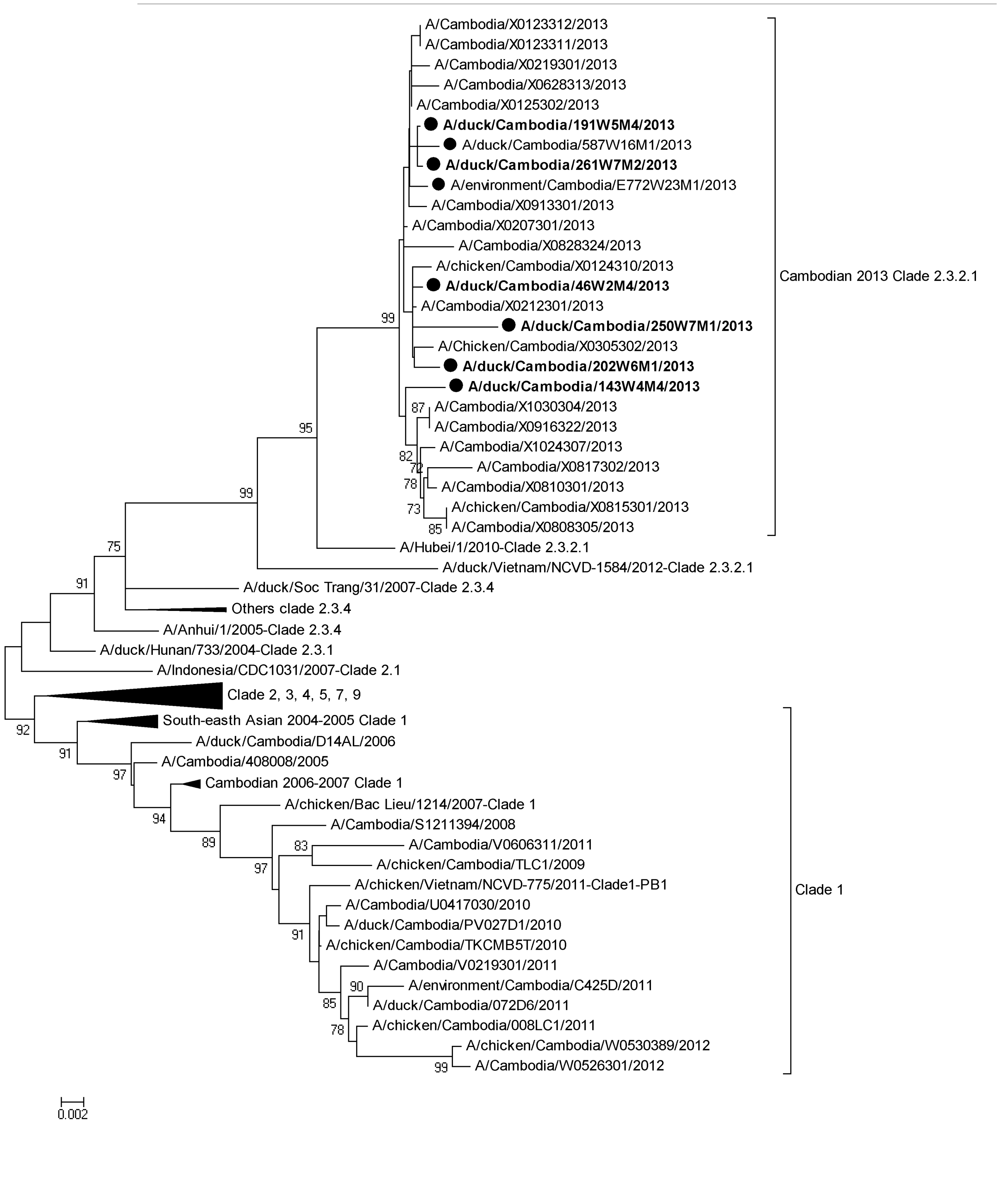

A/Cambodia/X0123312/2013
A/Cambodia/X0123311/2013
A/Cambodia/X0219301/2013
A/Cambodia/X0628313/2013
A/Cambodia/X0125302/2013
A/duck/Cambodia/191W5M4/2013
A/duck/Cambodia/587W16M1/2013
A/duck/Cambodia/261W7M2/2013
A/environment/Cambodia/E772W23M1/2013
A/Cambodia/X0913301/2013
A/Cambodia/X0207301/2013
A/Cambodia/X0828324/2013
A/chicken/Cambodia/X0124310/2013
A/duck/Cambodia/46W2M4/2013
A/Cambodia/X0212301/2013
A/duck/Cambodia/250W7M1/2013
A/Chicken/Cambodia/X0305302/2013
A/duck/Cambodia/202W6M1/2013
A/duck/Cambodia/143W4M4/2013
A/Cambodia/X1030304/2013
A/Cambodia/X0916322/2013
A/Cambodia/X1024307/2013
A/Cambodia/X0817302/2013
A/Cambodia/X0810301/2013
A/chicken/Cambodia/X0815301/2013
A/Cambodia/X0808305/2013
A/Hubei/1/2010-Clade 2.3.2.1
A/duck/Vietnam/NCVD-1584/2012-Clade 2.3.2.1
A/duck/Soc Trang/31/2007-Clade 2.3.4
Others clade 2.3.4
A/Anhui/1/2005-Clade 2.3.4
A/duck/Hunan/733/2004-Clade 2.3.1
A/Indonesia/CDC1031/2007-Clade 2.1
Clade 2, 3, 4, 5, 7, 9
South-easth Asian 2004-2005 Clade 1
A/duck/Cambodia/D14AL/2006
A/Cambodia/408008/2005
Cambodian 2006-2007 Clade 1
A/chicken/Bac Lieu/1214/2007-Clade 1
A/Cambodia/S1211394/2008
A/Cambodia/V0606311/2011
A/chicken/Cambodia/TLC1/2009
A/chicken/Vietnam/NCVD-775/2011-Clade1-PB1
A/Cambodia/U0417030/2010
A/duck/Cambodia/PV027D1/2010
A/chicken/Cambodia/TKCMB5T/2010
A/Cambodia/V0219301/2011
A/environment/Cambodia/C425D/2011
A/duck/Cambodia/072D6/2011
A/chicken/Cambodia/008LC1/2011
A/chicken/Cambodia/W0530389/2012
A/Cambodia/W0526301/2012
Cambodian 2013 Clade 2.3.2.1
Clade 1
99
95
99
75
91
92
91
97
94
89
97
83
91
90
85
78
99
87
82
72
78
73
85
0.002

PA

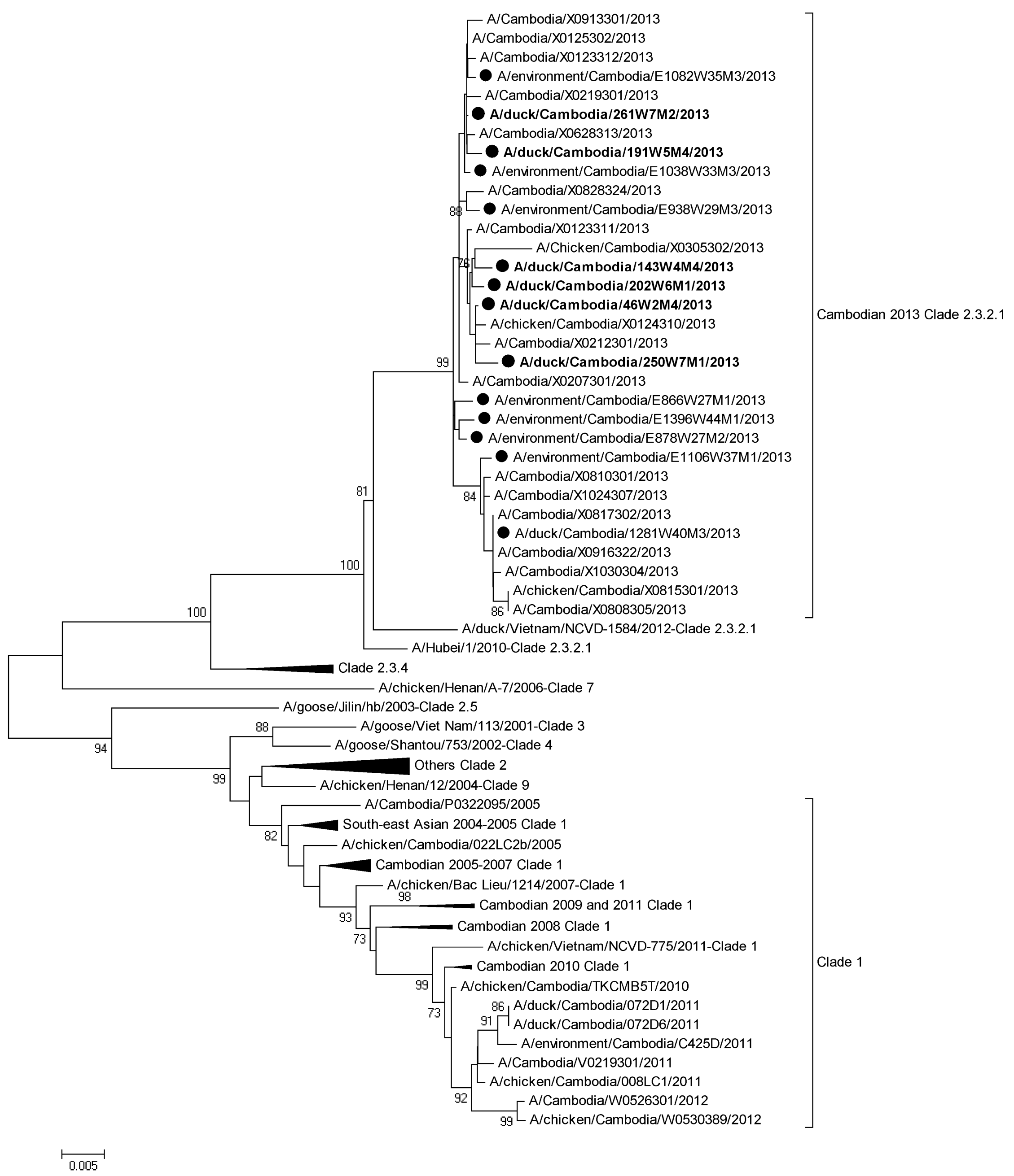

A/Cambodia/X0913301/2013
A/Cambodia/X0125302/2013
A/Cambodia/X0123312/2013
A/environment/Cambodia/E1082W35M3/2013
A/Cambodia/X0219301/2013
A/duck/Cambodia/261W7M2/2013
A/Cambodia/X0628313/2013
A/duck/Cambodia/191W5M4/2013
A/environment/Cambodia/E1038W33M3/2013
A/Cambodia/X0828324/2013
A/environment/Cambodia/E938W29M3/2013
A/Cambodia/X0123311/2013
A/Chicken/Cambodia/X0305302/2013
A/duck/Cambodia/143W4M4/2013
A/duck/Cambodia/202W6M1/2013
A/duck/Cambodia/46W2M4/2013
A/chicken/Cambodia/X0124310/2013
A/Cambodia/X0212301/2013
A/duck/Cambodia/250W7M1/2013
A/Cambodia/X0207301/2013
A/environment/Cambodia/E866W27M1/2013
A/environment/Cambodia/E1396W44M1/2013
A/environment/Cambodia/E878W27M2/2013
A/environment/Cambodia/E1106W37M1/2013
A/Cambodia/X0810301/2013
A/Cambodia/X1024307/2013
A/Cambodia/X0817302/2013
A/duck/Cambodia/1281W40M3/2013
A/Cambodia/X0916322/2013
A/Cambodia/X1030304/2013
A/chicken/Cambodia/X0815301/2013
A/Cambodia/X0808305/2013
A/duck/Vietnam/NCVD-1584/2012-Clade 2.3.2.1
A/Hubei/1/2010-Clade 2.3.2.1
Clade 2.3.4
A/chicken/Henan/A-7/2006-Clade 7
A/goose/Jilin/hb/2003-Clade 2.5
A/goose/Viet Nam/113/2001-Clade 3
A/goose/Shantou/753/2002-Clade 4
Others Clade 2
A/chicken/Henan/12/2004-Clade 9
A/Cambodia/P0322095/2005
South-east Asian 2004-2005 Clade 1
A/chicken/Cambodia/022LC2b/2005
Cambodian 2005-2007 Clade 1
A/chicken/Bac Lieu/1214/2007-Clade 1
Cambodian 2009 and 2011 Clade 1
Cambodian 2008 Clade 1
A/chicken/Vietnam/NCVD-775/2011-Clade 1
Cambodian 2010 Clade 1
A/chicken/Cambodia/TKCMB5T/2010
A/duck/Cambodia/072D1/2011
A/duck/Cambodia/072D6/2011
A/environment/Cambodia/C425D/2011
A/Cambodia/V0219301/2011
A/chicken/Cambodia/008LC1/2011
A/Cambodia/W0526301/2012
A/chicken/Cambodia/W0530389/2012
Cambodian 2013 Clade 2.3.2.1
Clade 1
88
76
99
81
84
100
86
100
94
88
99
82
98
93
73
99
73
86
91
92
99
0.005

NP

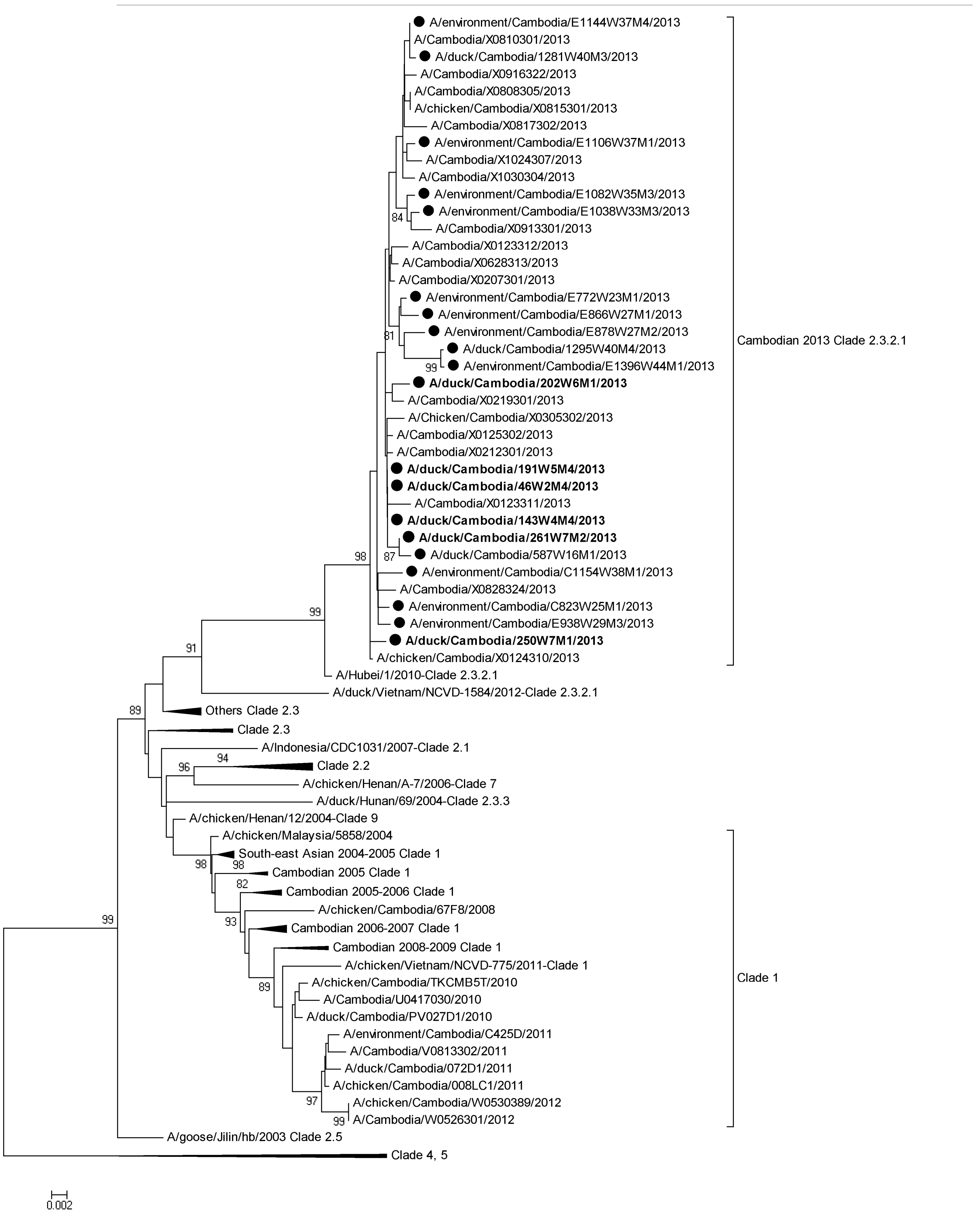
A/environment/Cambodia/E1144W37M4/2013
A/Cambodia/X0810301/2013
A/duck/Cambodia/1281W40M3/2013
A/Cambodia/X0916322/2013
A/Cambodia/X0808305/2013
A/chicken/Cambodia/X0815301/2013
A/Cambodia/X0817302/2013
A/environment/Cambodia/E1106W37M1/2013
A/Cambodia/X1024307/2013
A/Cambodia/X1030304/2013
A/environment/Cambodia/E1082W35M3/2013
84
A/environment/Cambodia/E1038W33M3/2013
A/Cambodia/X0913301/2013
A/Cambodia/X0123312/2013
A/Cambodia/X0628313/2013
A/Cambodia/X0207301/2013
A/environment/Cambodia/E772W23M1/2013
A/environment/Cambodia/E866W27M1/2013
81
A/environment/Cambodia/E878W27M2/2013
A/duck/Cambodia/1295W40M4/2013
99
A/environment/Cambodia/E1396W44M1/2013
A/duck/Cambodia/202W6M1/2013
A/Cambodia/X0219301/2013
A/Chicken/Cambodia/X0305302/2013
A/Cambodia/X0125302/2013
A/Cambodia/X0212301/2013
A/duck/Cambodia/191W5M4/2013
A/duck/Cambodia/46W2M4/2013
A/Cambodia/X0123311/2013
A/duck/Cambodia/143W4M4/2013
A/duck/Cambodia/261W7M2/2013
98
87
A/duck/Cambodia/587W16M1/2013
A/environment/Cambodia/C1154W38M1/2013
A/Cambodia/X0828324/2013
99
A/environment/Cambodia/C823W25M1/2013
A/environment/Cambodia/E938W29M3/2013
A/duck/Cambodia/250W7M1/2013
91
A/chicken/Cambodia/X0124310/2013
A/Hubei/1/2010-Clade 2.3.2.1
A/duck/Vietnam/NCVD-1584/2012-Clade 2.3.2.1
89
Others Clade 2.3
Clade 2.3
A/Indonesia/CDC1031/2007-Clade 2.1
94
96
Clade 2.2
A/chicken/Henan/A-7/2006-Clade 7
A/duck/Hunan/69/2004-Clade 2.3.3
A/chicken/Henan/12/2004-Clade 9
A/chicken/Malaysia/5858/2004
South-east Asian 2004-2005 Clade 1
98
98
Cambodian 2005 Clade 1
82
Cambodian 2005-2006 Clade 1
A/chicken/Cambodia/67F8/2008
99
93
Cambodian 2006-2007 Clade 1
Cambodian 2008-2009 Clade 1
A/chicken/Vietnam/NCVD-775/2011-Clade 1
A/chicken/Cambodia/TKCMB5T/2010
89
A/Cambodia/U0417030/2010
A/duck/Cambodia/PV027D1/2010
A/environment/Cambodia/C425D/2011
A/Cambodia/V0813302/2011
A/duck/Cambodia/072D1/2011
A/chicken/Cambodia/008LC1/2011
97
A/chicken/Cambodia/W0530389/2012
99
A/Cambodia/W0526301/2012
A/goose/Jilin/hb/2003 Clade 2.5
Clade 4, 5
0.002
Cambodian 2013 Clade 2.3.2.1
Clade 1

M

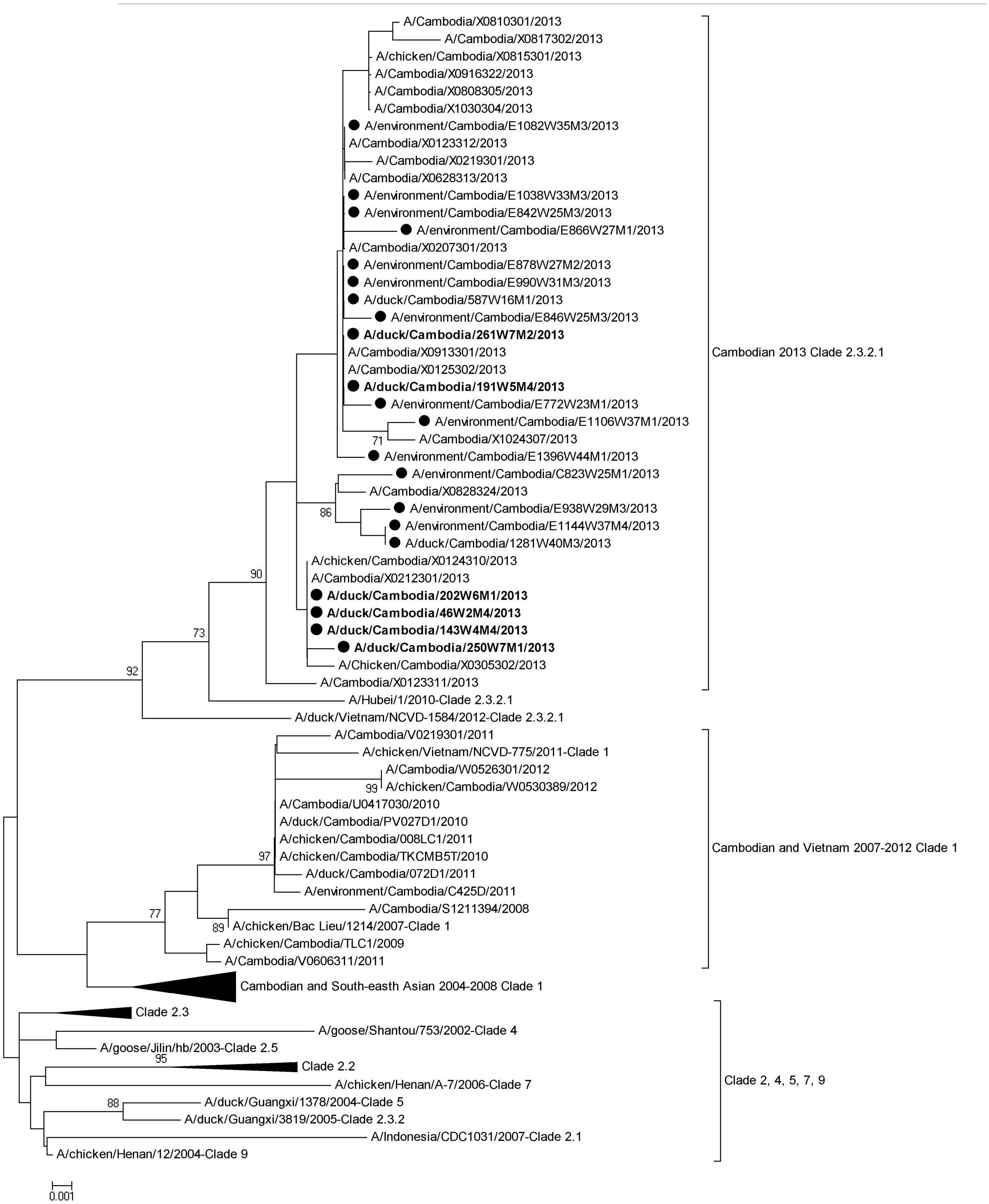
A/Cambodia/X0810301/2013
A/Cambodia/X0817302/2013
A/chicken/Cambodia/X0815301/2013
A/Cambodia/X0916322/2013
A/Cambodia/X0808305/2013
A/Cambodia/X1030304/2013
A/environment/Cambodia/E1082W35M3/2013
A/Cambodia/X0123312/2013
A/Cambodia/X0219301/2013
A/Cambodia/X0628313/2013
A/environment/Cambodia/E1038W33M3/2013
A/environment/Cambodia/E842W25M3/2013
A/environment/Cambodia/E866W27M1/2013
A/Cambodia/X0207301/2013
A/environment/Cambodia/E878W27M2/2013
A/environment/Cambodia/E990W31M3/2013
A/duck/Cambodia/587W16M1/2013
A/environment/Cambodia/E846W25M3/2013
A/duck/Cambodia/261W7M2/2013
A/Cambodia/X0913301/2013
A/Cambodia/X0125302/2013
A/duck/Cambodia/191W5M4/2013
A/environment/Cambodia/E772W23M1/2013
A/environment/Cambodia/E1106W37M1/2013
A/Cambodia/X1024307/2013
71
A/environment/Cambodia/E1396W44M1/2013
A/environment/Cambodia/C823W25M1/2013
A/Cambodia/X0828324/2013
86
A/environment/Cambodia/E938W29M3/2013
A/environment/Cambodia/E1144W37M4/2013
A/duck/Cambodia/1281W40M3/2013
A/chicken/Cambodia/X0124310/2013
A/Cambodia/X0212301/2013
90
A/duck/Cambodia/202W6M1/2013
A/duck/Cambodia/46W2M4/2013
73
A/duck/Cambodia/143W4M4/2013
A/duck/Cambodia/250W7M1/2013
A/Chicken/Cambodia/X0305302/2013
92
A/Cambodia/X0123311/2013
A/Hubei/1/2010-Clade 2.3.2.1
A/duck/Vietnam/NCVD-1584/2012-Clade 2.3.2.1
Cambodian 2013 Clade 2.3.2.1
A/Cambodia/V0219301/2011
A/chicken/Vietnam/NCVD-775/2011-Clade 1
A/Cambodia/W0526301/2012
99
A/chicken/Cambodia/W0530389/2012
A/Cambodia/U0417030/2010
A/duck/Cambodia/PV027D1/2010
A/chicken/Cambodia/008LC1/2011
97
A/chicken/Cambodia/TKCMB5T/2010
A/duck/Cambodia/072D1/2011
A/environment/Cambodia/C425D/2011
77
A/Cambodia/S1211394/2008
89
A/chicken/Bac Lieu/1214/2007-Clade 1
A/chicken/Cambodia/TLC1/2009
A/Cambodia/V0606311/2011
Cambodian and Vietnam 2007-2012 Clade 1
Cambodian and South-easth Asian 2004-2008 Clade 1
Clade 2.3
A/goose/Shantou/753/2002-Clade 4
A/goose/Jilin/hb/2003-Clade 2.5
95
Clade 2.2
A/chicken/Henan/A-7/2006-Clade 7
88
A/duck/Guangxi/1378/2004-Clade 5
A/duck/Guangxi/3819/2005-Clade 2.3.2
A/Indonesia/CDC1031/2007-Clade 2.1
A/chicken/Henan/12/2004-Clade 9
Clade 2, 4, 5, 7, 9
0.001

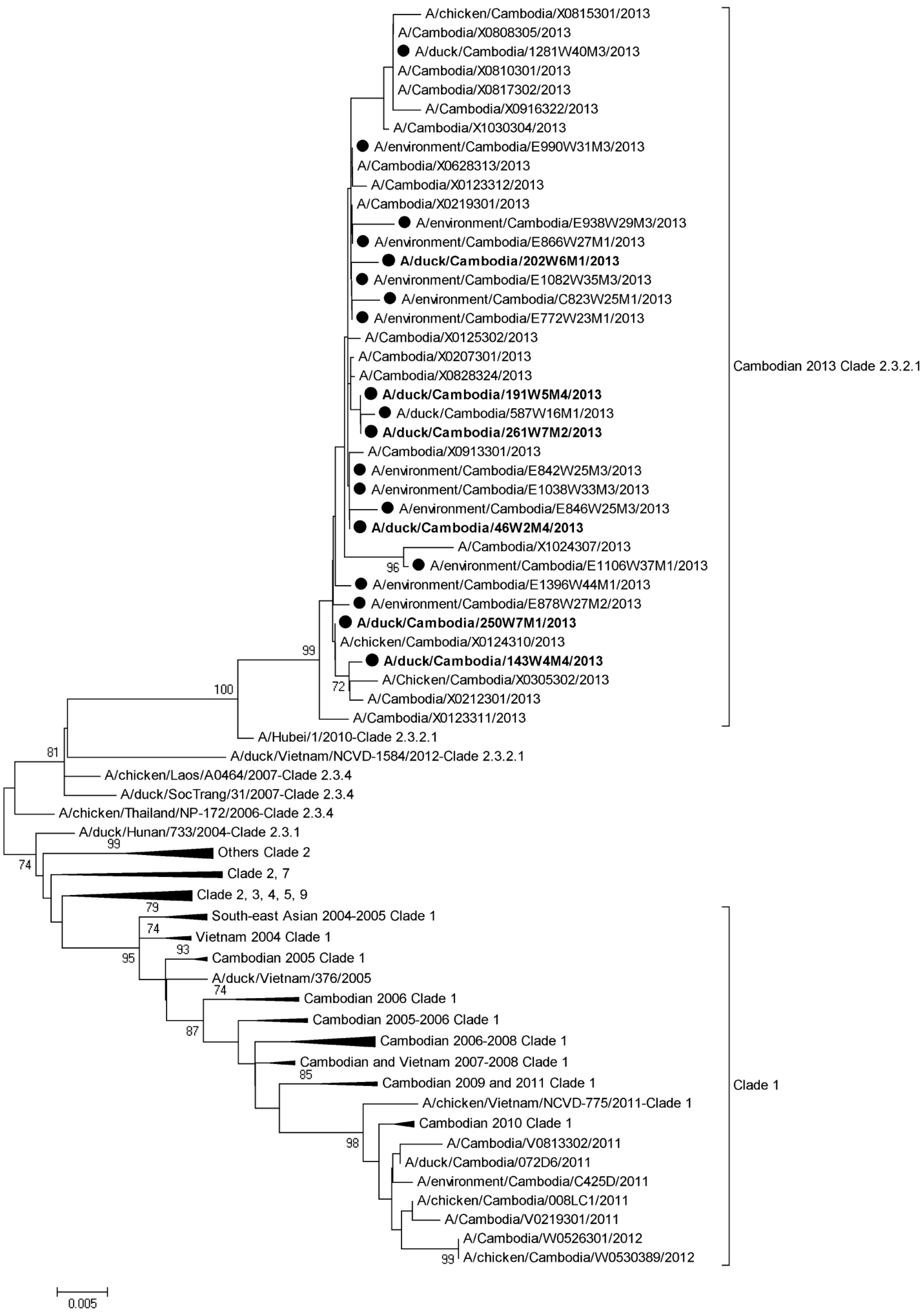

Supplementary Figure S1 Neighbor-joining phylogenetic trees of the PB2, PB1, PA, NP, M and NS genes of highly pathogenic avian influenza A/H5N1 viruses detected during live bird market surveillance in Cambodia. Viruses collected during the present market study are denoted by a black circle. Viruses for which the full genome is available are denoted in bold. The phylogenetic trees were constructed in MEGA5. Bootstraps greater than 70 generated from 1,000 replicates are shown at branch nodes. The scale bar represents the number of nucleotide substitutions per site.
