# Supplementary material for: Intense circulation of A/H5N1 and other avian influenza viruses in Cambodian live-bird markets with serological evidence of sub-clinical human infections
Source: Emerg Microbes Infect. 2016 Jul 20;5(7):e70–. doi: 10.1038/emi.2016.69 (PMC5141262; doi:10.1038/emi.2016.69)
Supplement: Supplementary Figure S2 [file emi201669x2.pdf]

H1

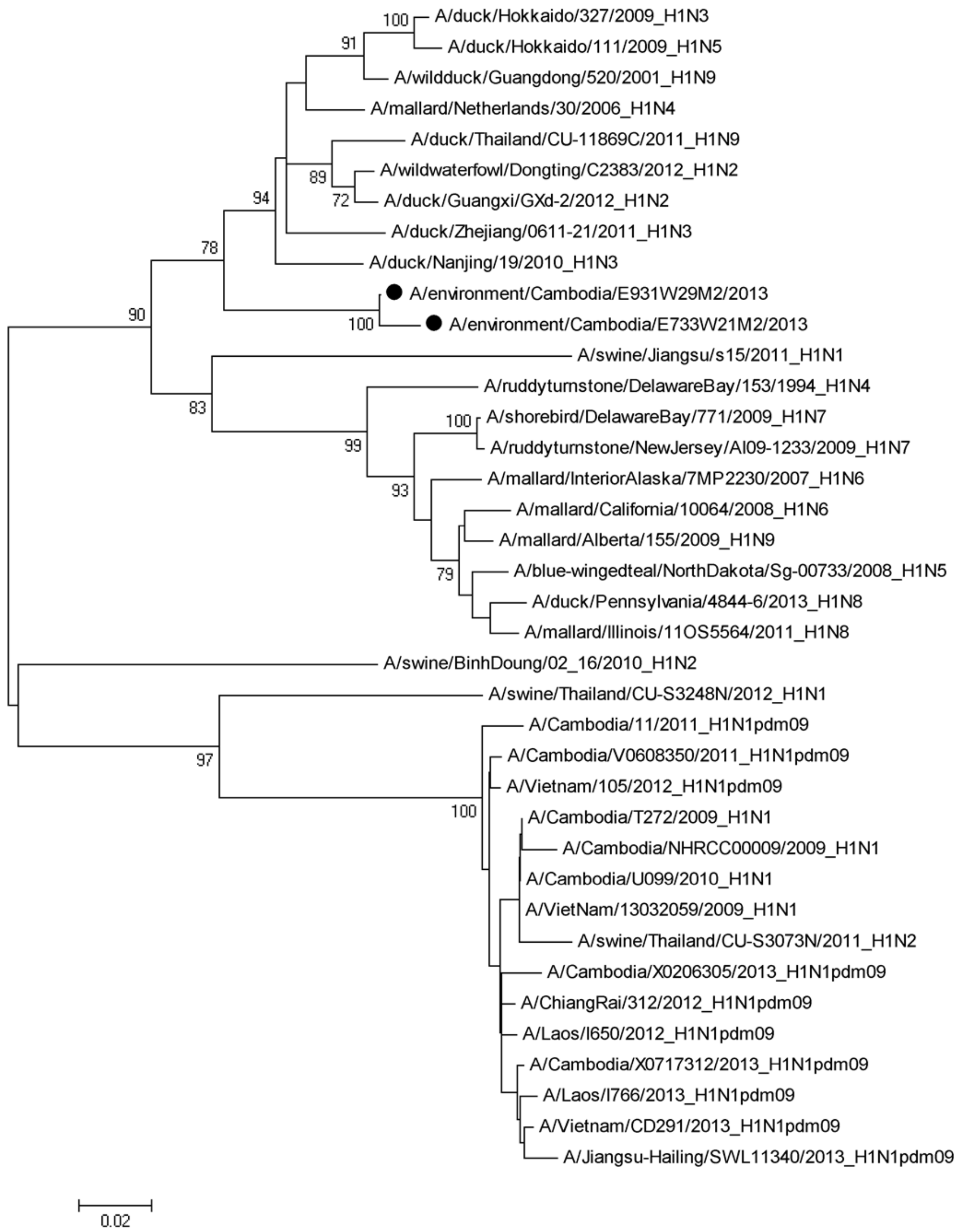

A/duck/Hokkaido/327/2009_H1N3
A/duck/Hokkaido/111/2009_H1N5
A/wildduck/Guangdong/520/2001_H1N9
A/mallard/Netherlands/30/2006_H1N4
A/duck/Thailand/CU-11869C/2011_H1N9
A/wildwaterfowl/Dongting/C2383/2012_H1N2
A/duck/Guangxi/GXd-2/2012_H1N2
A/duck/Zhejiang/0611-21/2011_H1N3
A/duck/Nanjing/19/2010_H1N3
A/environment/Cambodia/E931W29M2/2013
A/environment/Cambodia/E733W21M2/2013
A/swine/Jiangsu/s15/2011_H1N1
A/ruddyturnstone/DelawareBay/153/1994_H1N4
A/shorebird/DelawareBay/771/2009_H1N7
A/ruddyturnstone/NewJersey/AI09-1233/2009_H1N7
A/mallard/InteriorAlaska/7MP2230/2007_H1N6
A/mallard/California/10064/2008_H1N6
A/mallard/Alberta/155/2009_H1N9
A/blue-wingedteal/NorthDakota/Sg-00733/2008_H1N5
A/duck/Pennsylvania/4844-6/2013_H1N8
A/mallard/Illinois/11OS5564/2011_H1N8
A/swine/BinhDoung/02_16/2010_H1N2
A/swine/Thailand/CU-S3248N/2012_H1N1
A/Cambodia/11/2011_H1N1pdm09
A/Cambodia/V0608350/2011_H1N1pdm09
A/Vietnam/105/2012_H1N1pdm09
A/Cambodia/T272/2009_H1N1
A/Cambodia/NHRCC00009/2009_H1N1
A/Cambodia/U099/2010_H1N1
A/VietNam/13032059/2009_H1N1
A/swine/Thailand/CU-S3073N/2011_H1N2
A/Cambodia/X0206305/2013_H1N1pdm09
A/ChiangRai/312/2012_H1N1pdm09
A/Laos/I650/2012_H1N1pdm09
A/Cambodia/X0717312/2013_H1N1pdm09
A/Laos/I766/2013_H1N1pdm09
A/Vietnam/CD291/2013_H1N1pdm09
A/Jiangsu-Hailing/SWL11340/2013_H1N1pdm09
100
91
89
72
94
78
100
90
83
99
100
93
79
97
100
0.02

H2

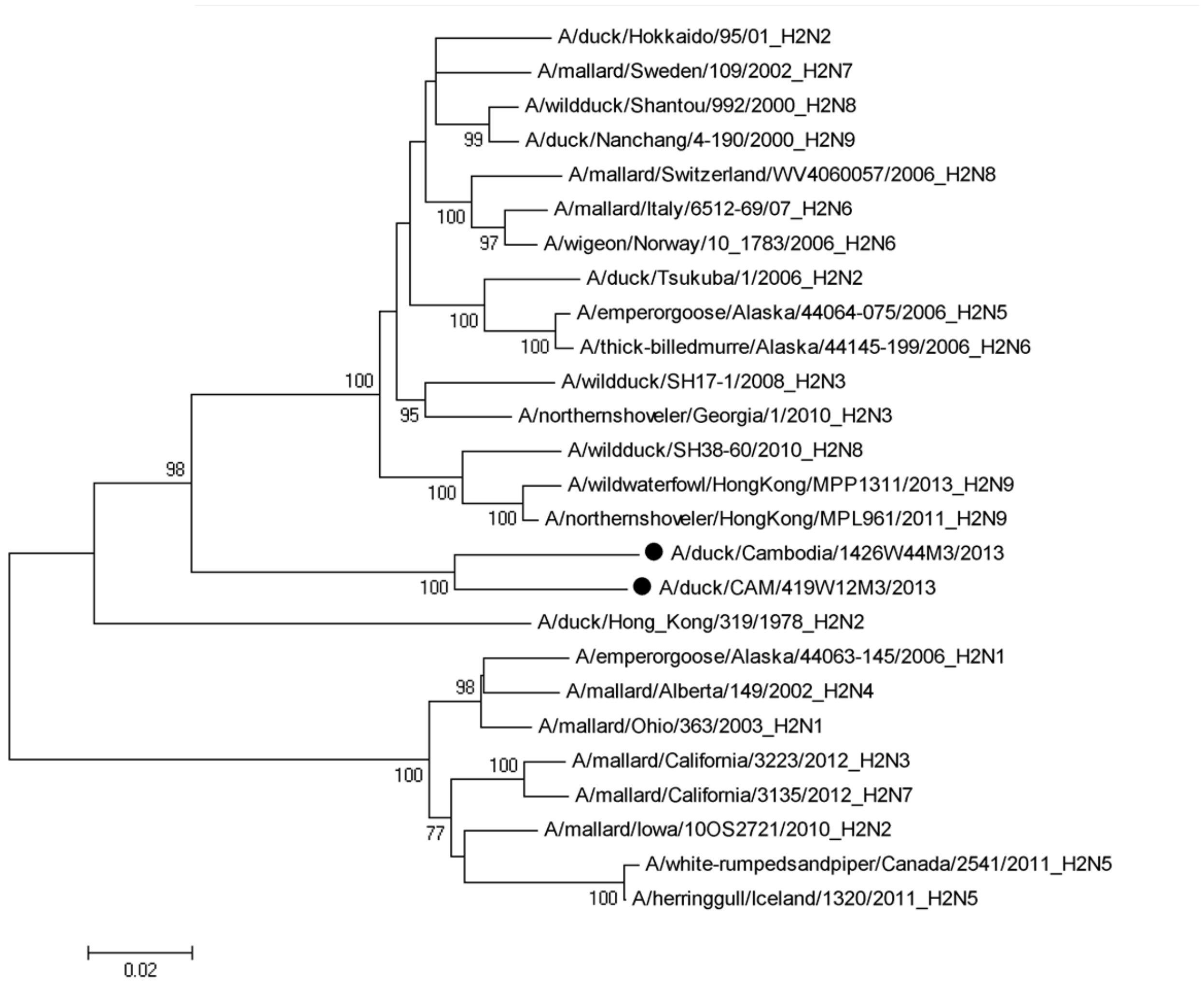

A/duck/Hokkaido/95/01_H2N2
A/mallard/Sweden/109/2002_H2N7
A/wildduck/Shantou/992/2000_H2N8
99
A/duck/Nanchang/4-190/2000_H2N9
A/mallard/Switzerland/WV4060057/2006_H2N8
100
A/mallard/Italy/6512-69/07_H2N6
97
A/wigeon/Norway/10_1783/2006_H2N6
A/duck/Tsukuba/1/2006_H2N2
100
A/emperorgoose/Alaska/44064-075/2006_H2N5
100
A/thick-billedmurre/Alaska/44145-199/2006_H2N6
100
A/wildduck/SH17-1/2008_H2N3
95
A/northernshoveler/Georgia/1/2010_H2N3
98
A/wildduck/SH38-60/2010_H2N8
100
A/wildwaterfowl/HongKong/MPP1311/2013_H2N9
100
A/northernshoveler/HongKong/MPL961/2011_H2N9
A/duck/Cambodia/1426W44M3/2013
100
A/duck/CAM/419W12M3/2013
A/duck/Hong_Kong/319/1978_H2N2
A/emperorgoose/Alaska/44063-145/2006_H2N1
98
A/mallard/Alberta/149/2002_H2N4
A/mallard/Ohio/363/2003_H2N1
100
100
A/mallard/California/3223/2012_H2N3
A/mallard/California/3135/2012_H2N7
77
A/mallard/Iowa/10OS2721/2010_H2N2
A/white-rumpedsandpiper/Canada/2541/2011_H2N5
100
A/herringgull/Iceland/1320/2011_H2N5
0.02

H3

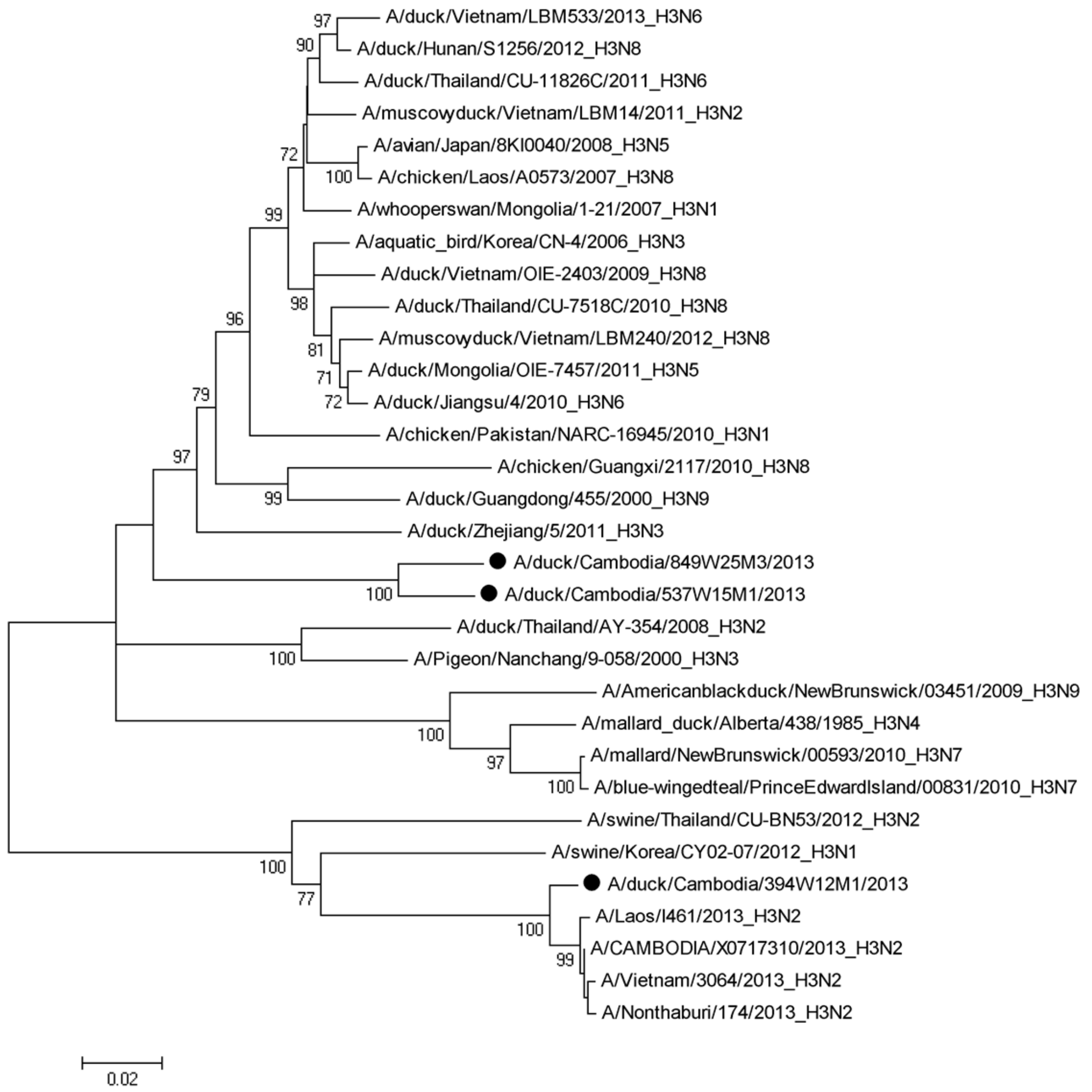

A/duck/Vietnam/LBM533/2013_H3N6
A/duck/Hunan/S1256/2012_H3N8
A/duck/Thailand/CU-11826C/2011_H3N6
A/muscovyduck/Vietnam/LBM14/2011_H3N2
A/avian/Japan/8KI0040/2008_H3N5
A/chicken/Laos/A0573/2007_H3N8
A/whooperswan/Mongolia/1-21/2007_H3N1
A/aquatic_bird/Korea/CN-4/2006_H3N3
A/duck/Vietnam/OIE-2403/2009_H3N8
A/duck/Thailand/CU-7518C/2010_H3N8
A/muscovyduck/Vietnam/LBM240/2012_H3N8
A/duck/Mongolia/OIE-7457/2011_H3N5
A/duck/Jiangsu/4/2010_H3N6
A/chicken/Pakistan/NARC-16945/2010_H3N1
A/chicken/Guangxi/2117/2010_H3N8
A/duck/Guangdong/455/2000_H3N9
A/duck/Zhejiang/5/2011_H3N3
A/duck/Cambodia/849W25M3/2013
A/duck/Cambodia/537W15M1/2013
A/duck/Thailand/AY-354/2008_H3N2
A/Pigeon/Nanchang/9-058/2000_H3N3
A/Americanblackduck/NewBrunswick/03451/2009_H3N9
A/mallard_duck/Alberta/438/1985_H3N4
A/mallard/NewBrunswick/00593/2010_H3N7
A/blue-wingedteal/PrinceEdwardIsland/00831/2010_H3N7
A/swine/Thailand/CU-BN53/2012_H3N2
A/swine/Korea/CY02-07/2012_H3N1
A/duck/Cambodia/394W12M1/2013
A/Laos/I461/2013_H3N2
A/CAMBODIA/X0717310/2013_H3N2
A/Vietnam/3064/2013_H3N2
A/Nonthaburi/174/2013_H3N2
97
90
72
100
99
98
81
71
72
96
79
97
99
100
100
100
97
100
100
77
100
99
0.02

H4

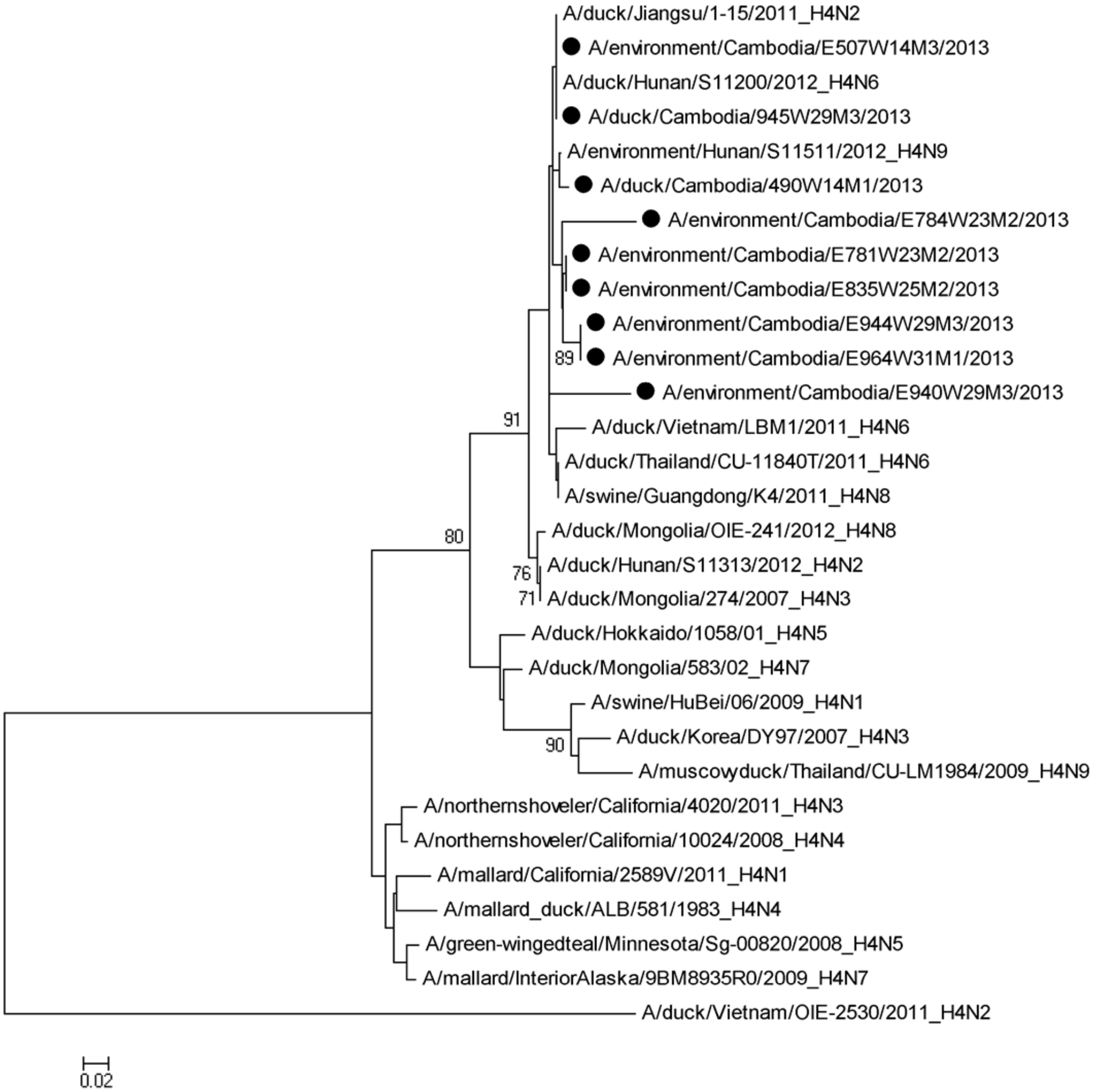
A/duck/Jiangsu/1-15/2011_H4N2
A/environment/Cambodia/E507W14M3/2013
A/duck/Hunan/S11200/2012_H4N6
A/duck/Cambodia/945W29M3/2013
A/environment/Hunan/S11511/2012_H4N9
A/duck/Cambodia/490W14M1/2013
A/environment/Cambodia/E784W23M2/2013
A/environment/Cambodia/E781W23M2/2013
A/environment/Cambodia/E835W25M2/2013
A/environment/Cambodia/E944W29M3/2013
89
A/environment/Cambodia/E964W31M1/2013
A/environment/Cambodia/E940W29M3/2013
91
A/duck/Vietnam/LBM1/2011_H4N6
A/duck/Thailand/CU-11840T/2011_H4N6
A/swine/Guangdong/K4/2011_H4N8
80
A/duck/Mongolia/OIE-241/2012_H4N8
76
A/duck/Hunan/S11313/2012_H4N2
71
A/duck/Mongolia/274/2007_H4N3
A/duck/Hokkaido/1058/01_H4N5
A/duck/Mongolia/583/02_H4N7
A/swine/HuBei/06/2009_H4N1
90
A/duck/Korea/DY97/2007_H4N3
A/muscovyduck/Thailand/CU-LM1984/2009_H4N9
A/northernshoveler/California/4020/2011_H4N3
A/northernshoveler/California/10024/2008_H4N4
A/mallard/California/2589V/2011_H4N1
A/mallard_duck/ALB/581/1983_H4N4
A/green-wingedteal/Minnesota/Sg-00820/2008_H4N5
A/mallard/InteriorAlaska/9BM8935R0/2009_H4N7
A/duck/Vietnam/OIE-2530/2011_H4N2
0.02

H6

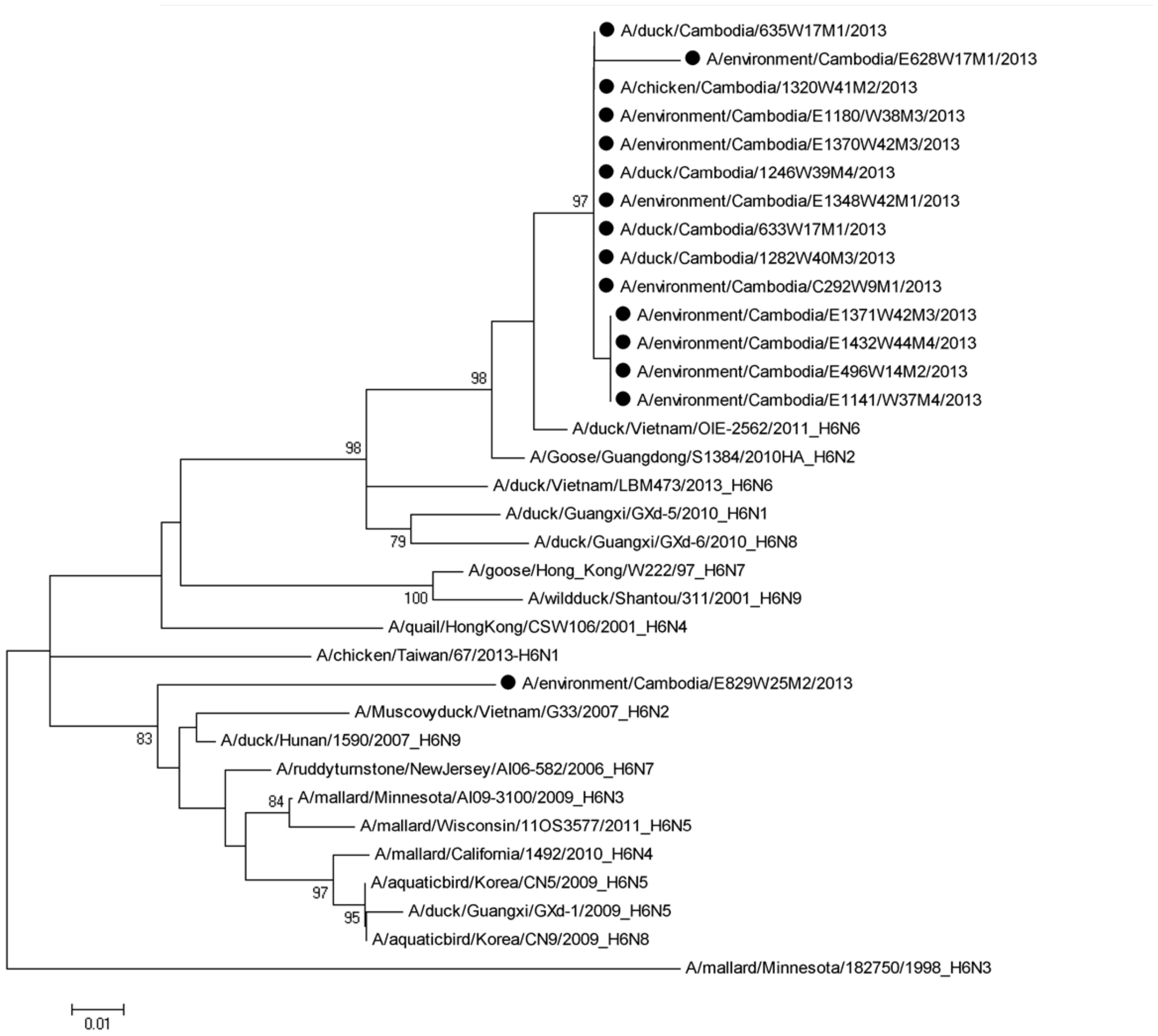

A/duck/Cambodia/635W17M1/2013
A/environment/Cambodia/E628W17M1/2013
A/chicken/Cambodia/1320W41M2/2013
A/environment/Cambodia/E1180/W38M3/2013
A/environment/Cambodia/E1370W42M3/2013
A/duck/Cambodia/1246W39M4/2013
97
A/environment/Cambodia/E1348W42M1/2013
A/duck/Cambodia/633W17M1/2013
A/duck/Cambodia/1282W40M3/2013
A/environment/Cambodia/C292W9M1/2013
A/environment/Cambodia/E1371W42M3/2013
A/environment/Cambodia/E1432W44M4/2013
A/environment/Cambodia/E496W14M2/2013
98
A/environment/Cambodia/E1141/W37M4/2013
A/duck/Vietnam/OIE-2562/2011_H6N6
98
A/Goose/Guangdong/S1384/2010HA_H6N2
A/duck/Vietnam/LBM473/2013_H6N6
A/duck/Guangxi/GXd-5/2010_H6N1
79
A/duck/Guangxi/GXd-6/2010_H6N8
A/goose/Hong_Kong/W222/97_H6N7
100
A/wildduck/Shantou/311/2001_H6N9
A/quail/HongKong/CSW106/2001_H6N4
A/chicken/Taiwan/67/2013-H6N1
A/environment/Cambodia/E829W25M2/2013
A/Muscovyduck/Vietnam/G33/2007_H6N2
83
A/duck/Hunan/1590/2007_H6N9
A/ruddyturnstone/NewJersey/AI06-582/2006_H6N7
84
A/mallard/Minnesota/AI09-3100/2009_H6N3
A/mallard/Wisconsin/11OS3577/2011_H6N5
A/mallard/California/1492/2010_H6N4
97
A/aquaticbird/Korea/CN5/2009_H6N5
95
A/duck/Guangxi/GXd-1/2009_H6N5
A/aquaticbird/Korea/CN9/2009_H6N8
A/mallard/Minnesota/182750/1998_H6N3
0.01

H7

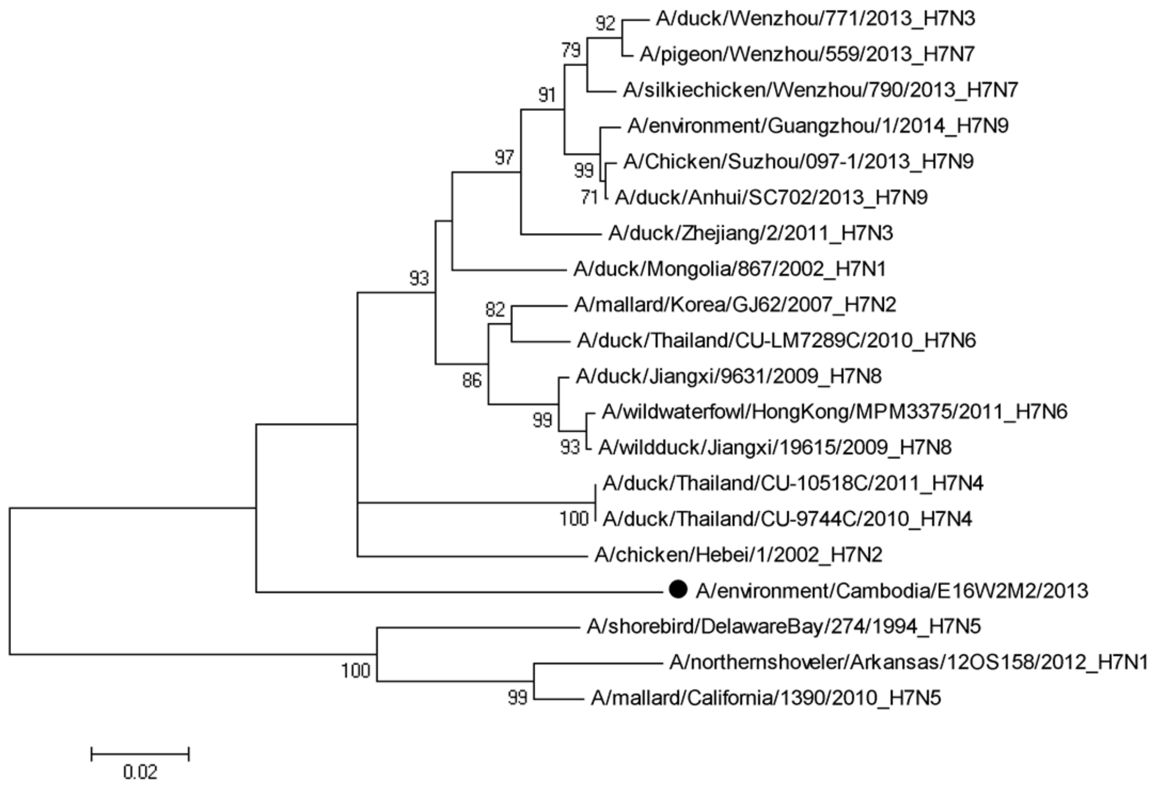
92
A/duck/Wenzhou/771/2013_H7N3
79
A/pigeon/Wenzhou/559/2013_H7N7
91
A/silkiechicken/Wenzhou/790/2013_H7N7
A/environment/Guangzhou/1/2014_H7N9
97
99
A/Chicken/Suzhou/097-1/2013_H7N9
71
A/duck/Anhui/SC702/2013_H7N9
A/duck/Zhejiang/2/2011_H7N3
A/duck/Mongolia/867/2002_H7N1
93
82
A/mallard/Korea/GJ62/2007_H7N2
A/duck/Thailand/CU-LM7289C/2010_H7N6
86
A/duck/Jiangxi/9631/2009_H7N8
99
A/wildwaterfowl/HongKong/MPM3375/2011_H7N6
93
A/wildduck/Jiangxi/19615/2009_H7N8
A/duck/Thailand/CU-10518C/2011_H7N4
100
A/duck/Thailand/CU-9744C/2010_H7N4
A/chicken/Hebei/1/2002_H7N2
A/environment/Cambodia/E16W2M2/2013
A/shorebird/DelawareBay/274/1994_H7N5
100
A/northernshoveler/Arkansas/12OS158/2012_H7N1
99
A/mallard/California/1390/2010_H7N5
0.02

H9

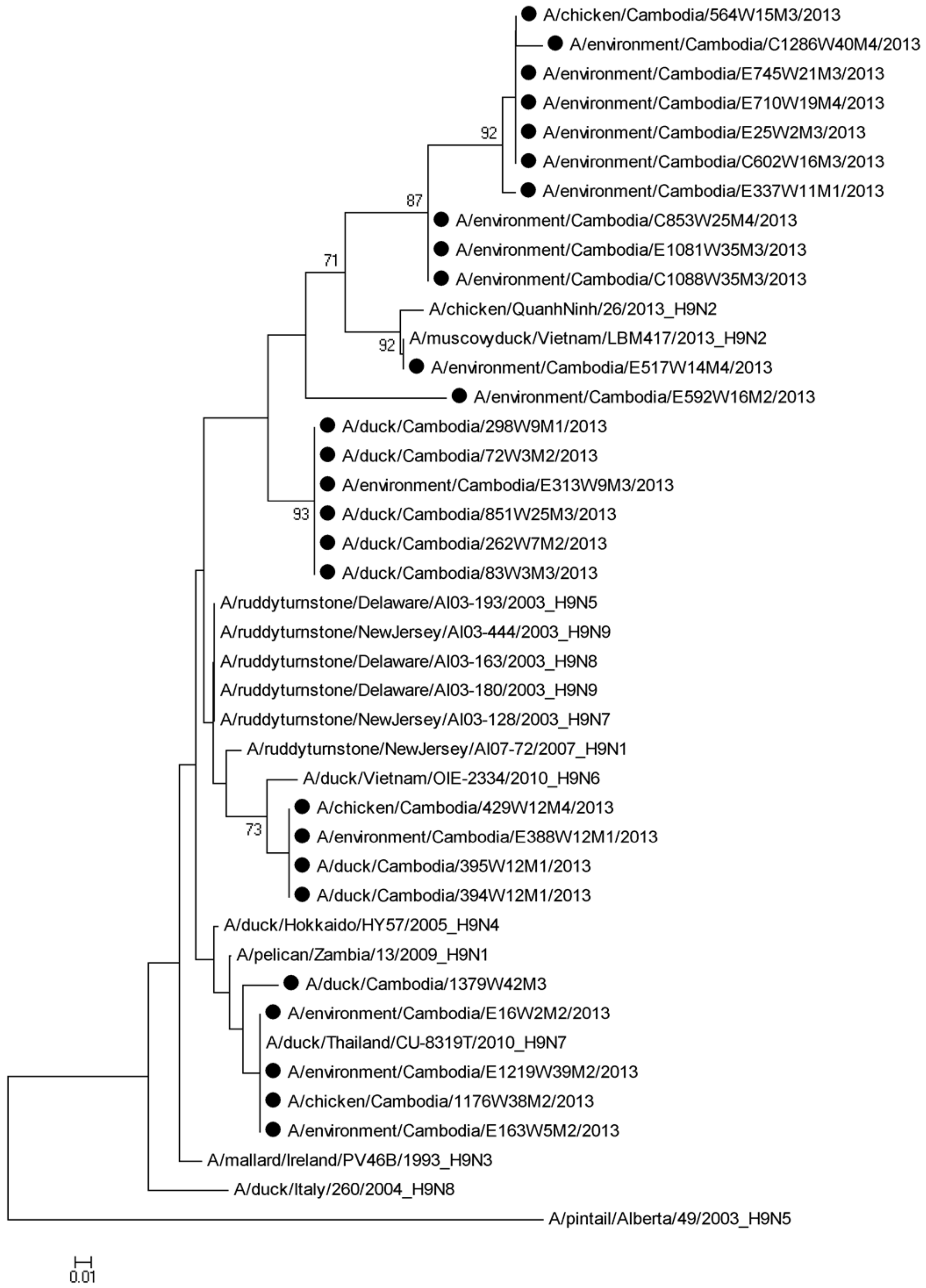
A/chicken/Cambodia/564W15M3/2013
A/environment/Cambodia/C1286W40M4/2013
A/environment/Cambodia/E745W21M3/2013
A/environment/Cambodia/E710W19M4/2013
A/environment/Cambodia/E25W2M3/2013
A/environment/Cambodia/C602W16M3/2013
A/environment/Cambodia/E337W11M1/2013
92
87
A/environment/Cambodia/C853W25M4/2013
A/environment/Cambodia/E1081W35M3/2013
A/environment/Cambodia/C1088W35M3/2013
71
A/chicken/QuanhNinh/26/2013_H9N2
A/muscovyduck/Vietnam/LBM417/2013_H9N2
92
A/environment/Cambodia/E517W14M4/2013
A/environment/Cambodia/E592W16M2/2013
A/duck/Cambodia/298W9M1/2013
A/duck/Cambodia/72W3M2/2013
A/environment/Cambodia/E313W9M3/2013
93
A/duck/Cambodia/851W25M3/2013
A/duck/Cambodia/262W7M2/2013
A/duck/Cambodia/83W3M3/2013
A/ruddyturnstone/Delaware/AI03-193/2003_H9N5
A/ruddyturnstone/NewJersey/AI03-444/2003_H9N9
A/ruddyturnstone/Delaware/AI03-163/2003_H9N8
A/ruddyturnstone/Delaware/AI03-180/2003_H9N9
A/ruddyturnstone/NewJersey/AI03-128/2003_H9N7
A/ruddyturnstone/NewJersey/AI07-72/2007_H9N1
A/duck/Vietnam/OIE-2334/2010_H9N6
A/chicken/Cambodia/429W12M4/2013
73
A/environment/Cambodia/E388W12M1/2013
A/duck/Cambodia/395W12M1/2013
A/duck/Cambodia/394W12M1/2013
A/duck/Hokkaido/HY57/2005_H9N4
A/pelican/Zambia/13/2009_H9N1
A/duck/Cambodia/1379W42M3
A/environment/Cambodia/E16W2M2/2013
A/duck/Thailand/CU-8319T/2010_H9N7
A/environment/Cambodia/E1219W39M2/2013
A/chicken/Cambodia/1176W38M2/2013
A/environment/Cambodia/E163W5M2/2013
A/mallard/Ireland/PV46B/1993_H9N3
A/duck/Italy/260/2004_H9N8
A/pintail/Alberta/49/2003_H9N5
0.01

H10

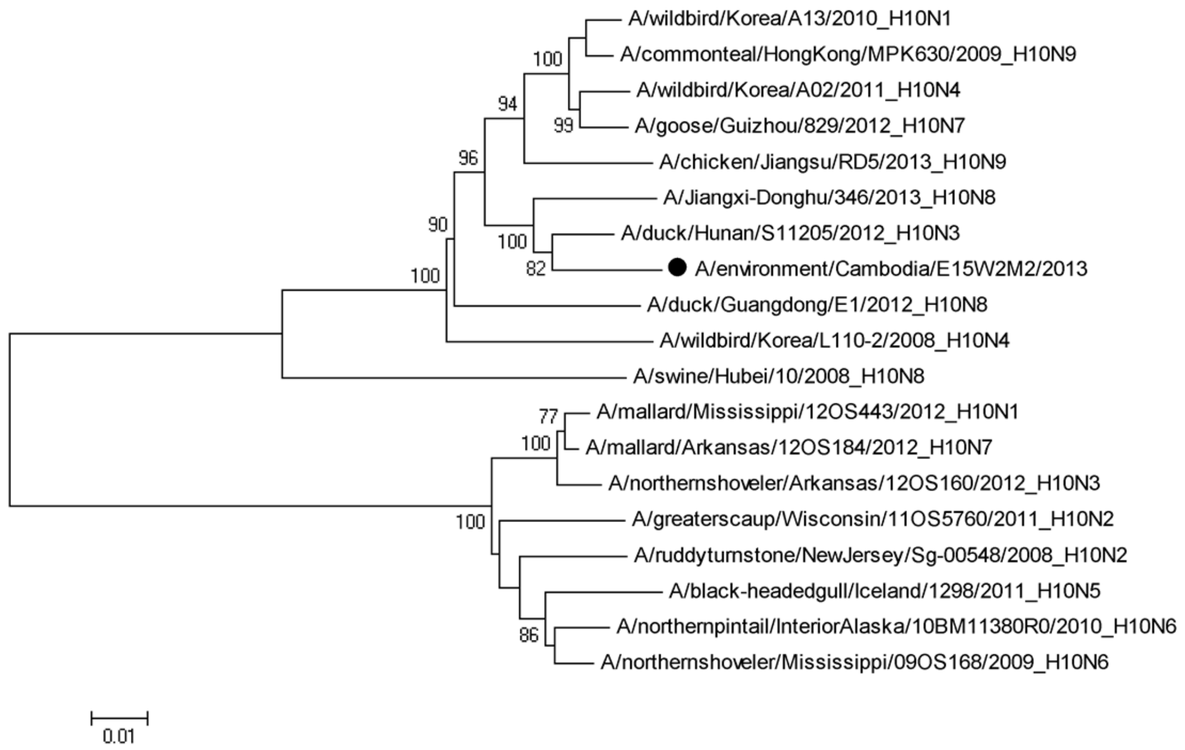
A/wildbird/Korea/A13/2010_H10N1
A/commonteal/HongKong/MPK630/2009_H10N9
A/wildbird/Korea/A02/2011_H10N4
A/goose/Guizhou/829/2012_H10N7
A/chicken/Jiangsu/RD5/2013_H10N9
A/Jiangxi-Donghu/346/2013_H10N8
A/duck/Hunan/S11205/2012_H10N3
A/environment/Cambodia/E15W2M2/2013
A/duck/Guangdong/E1/2012_H10N8
A/wildbird/Korea/L110-2/2008_H10N4
A/swine/Hubei/10/2008_H10N8
A/mallard/Mississippi/12OS443/2012_H10N1
A/mallard/Arkansas/12OS184/2012_H10N7
A/northernshoveler/Arkansas/12OS160/2012_H10N3
A/greaterscaup/Wisconsin/11OS5760/2011_H10N2
A/ruddyturnstone/NewJersey/Sg-00548/2008_H10N2
A/black-headedgull/Iceland/1298/2011_H10N5
A/northernpintail/InteriorAlaska/10BM11380R0/2010_H10N6
A/northernshoveler/Mississippi/09OS168/2009_H10N6
100
99
94
96
90
100
82
100
77
100
100
86
0.01

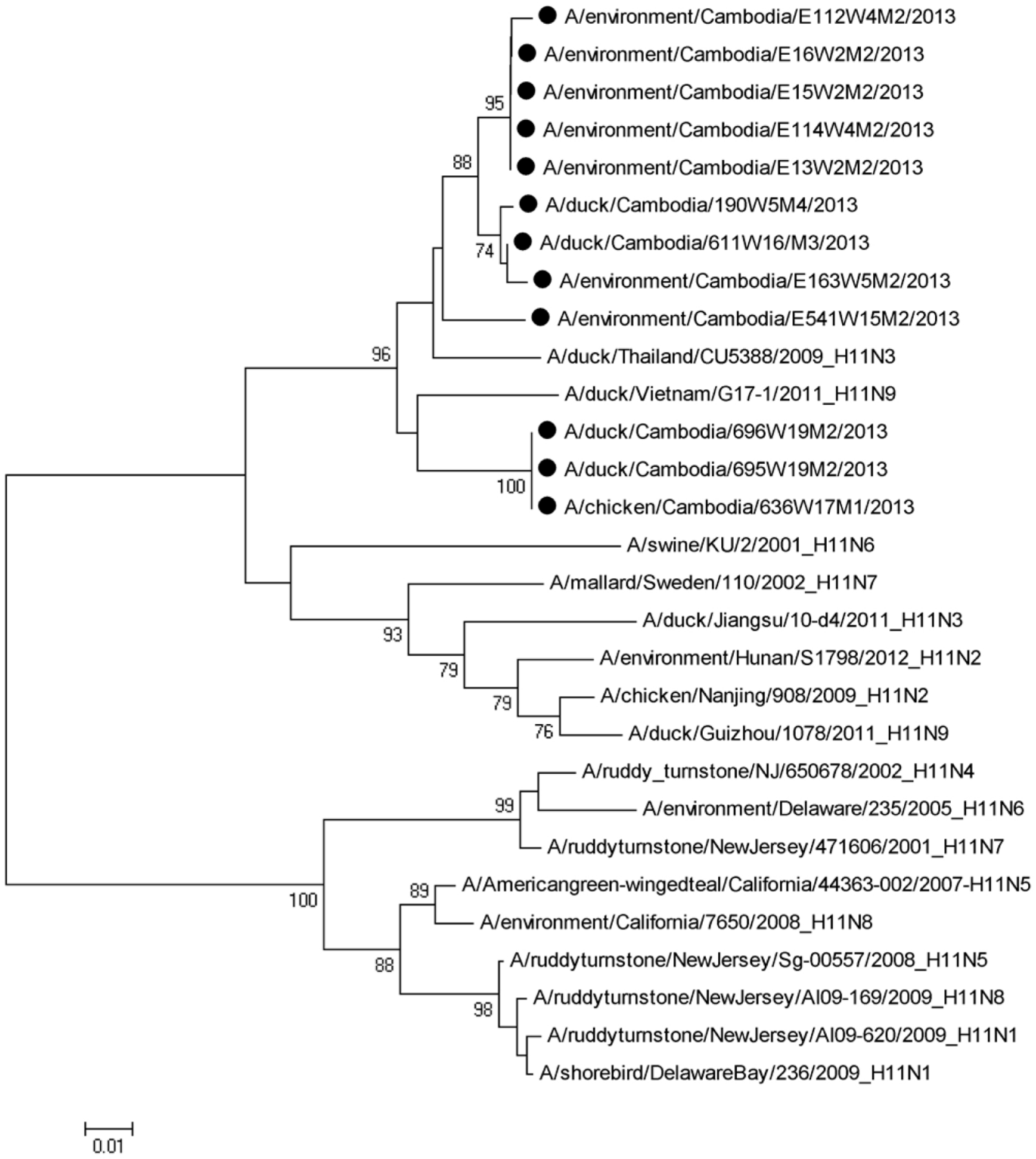

Supplementary Figure S2 Neighbor-joining phylogenetic trees of the haemagglutinin (HA) genes of avian influenza viruses detected during live bird market surveillance in Cambodia. Viruses collected during the present market study are denoted by a black circle. The phylogenetic trees were constructed in MEGA5. Bootstraps greater than 70 generated from 1,000 replicates are shown at branch nodes. The scale bar represents the number of nucleotide substitutions per site.
