# Supplementary material for: Intense circulation of A/H5N1 and other avian influenza viruses in Cambodian live-bird markets with serological evidence of sub-clinical human infections
Source: Emerg Microbes Infect. 2016 Jul 20;5(7):e70–. doi: 10.1038/emi.2016.69 (PMC5141262; doi:10.1038/emi.2016.69)
Supplement: Supplementary Figure S3 [file emi201669x3.pdf]

N2

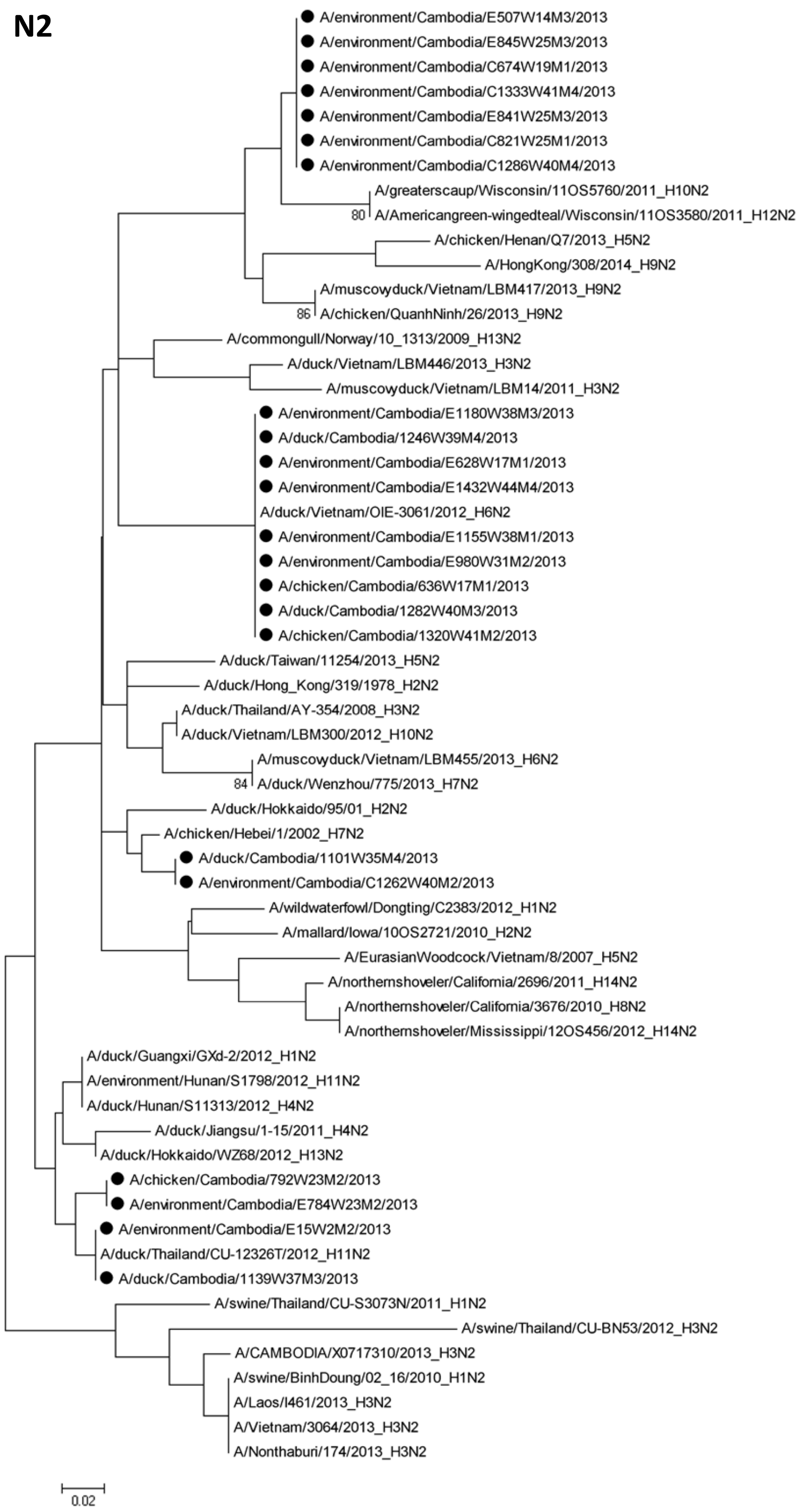
A/environment/Cambodia/E507W14M3/2013
A/environment/Cambodia/E845W25M3/2013
A/environment/Cambodia/C674W19M1/2013
A/environment/Cambodia/C1333W41M4/2013
A/environment/Cambodia/E841W25M3/2013
A/environment/Cambodia/C821W25M1/2013
A/environment/Cambodia/C1286W40M4/2013
A/greaterscaup/Wisconsin/11OS5760/2011_H10N2
80
A/Americangreen-wingedteal/Wisconsin/11OS3580/2011_H12N2
A/chicken/Henan/Q7/2013_H5N2
A/HongKong/308/2014_H9N2
A/muscovyduck/Vietnam/LBM417/2013_H9N2
86
A/chicken/QuanhNinh/26/2013_H9N2
A/commongull/Norway/10_1313/2009_H13N2
A/duck/Vietnam/LBM446/2013_H3N2
A/muscovyduck/Vietnam/LBM14/2011_H3N2
A/environment/Cambodia/E1180W38M3/2013
A/duck/Cambodia/1246W39M4/2013
A/environment/Cambodia/E628W17M1/2013
A/environment/Cambodia/E1432W44M4/2013
A/duck/Vietnam/OIE-3061/2012_H6N2
A/environment/Cambodia/E1155W38M1/2013
A/environment/Cambodia/E980W31M2/2013
A/chicken/Cambodia/636W17M1/2013
A/duck/Cambodia/1282W40M3/2013
A/chicken/Cambodia/1320W41M2/2013
A/duck/Taiwan/11254/2013_H5N2
A/duck/Hong_Kong/319/1978_H2N2
A/duck/Thailand/AY-354/2008_H3N2
A/duck/Vietnam/LBM300/2012_H10N2
A/muscovyduck/Vietnam/LBM455/2013_H6N2
84
A/duck/Wenzhou/775/2013_H7N2
A/duck/Hokkaido/95/01_H2N2
A/chicken/Hebei/1/2002_H7N2
A/duck/Cambodia/1101W35M4/2013
A/environment/Cambodia/C1262W40M2/2013
A/wildwaterfowl/Dongting/C2383/2012_H1N2
A/mallard/Iowa/10OS2721/2010_H2N2
A/EurasianWoodcock/Vietnam/8/2007_H5N2
A/northernshoveler/California/2696/2011_H14N2
A/northernshoveler/California/3676/2010_H8N2
A/northernshoveler/Mississippi/12OS456/2012_H14N2
A/duck/Guangxi/GXd-2/2012_H1N2
A/environment/Hunan/S1798/2012_H11N2
A/duck/Hunan/S11313/2012_H4N2
A/duck/Jiangsu/1-15/2011_H4N2
A/duck/Hokkaido/WZ68/2012_H13N2
A/chicken/Cambodia/792W23M2/2013
A/environment/Cambodia/E784W23M2/2013
A/environment/Cambodia/E15W2M2/2013
A/duck/Thailand/CU-12326T/2012_H11N2
A/duck/Cambodia/1139W37M3/2013
A/swine/Thailand/CU-S3073N/2011_H1N2
A/swine/Thailand/CU-BN53/2012_H3N2
A/CAMBODIA/X0717310/2013_H3N2
A/swine/BinhDoung/02_16/2010_H1N2
A/Laos/I461/2013_H3N2
A/Vietnam/3064/2013_H3N2
A/Nonthaburi/174/2013_H3N2
0.02

N3

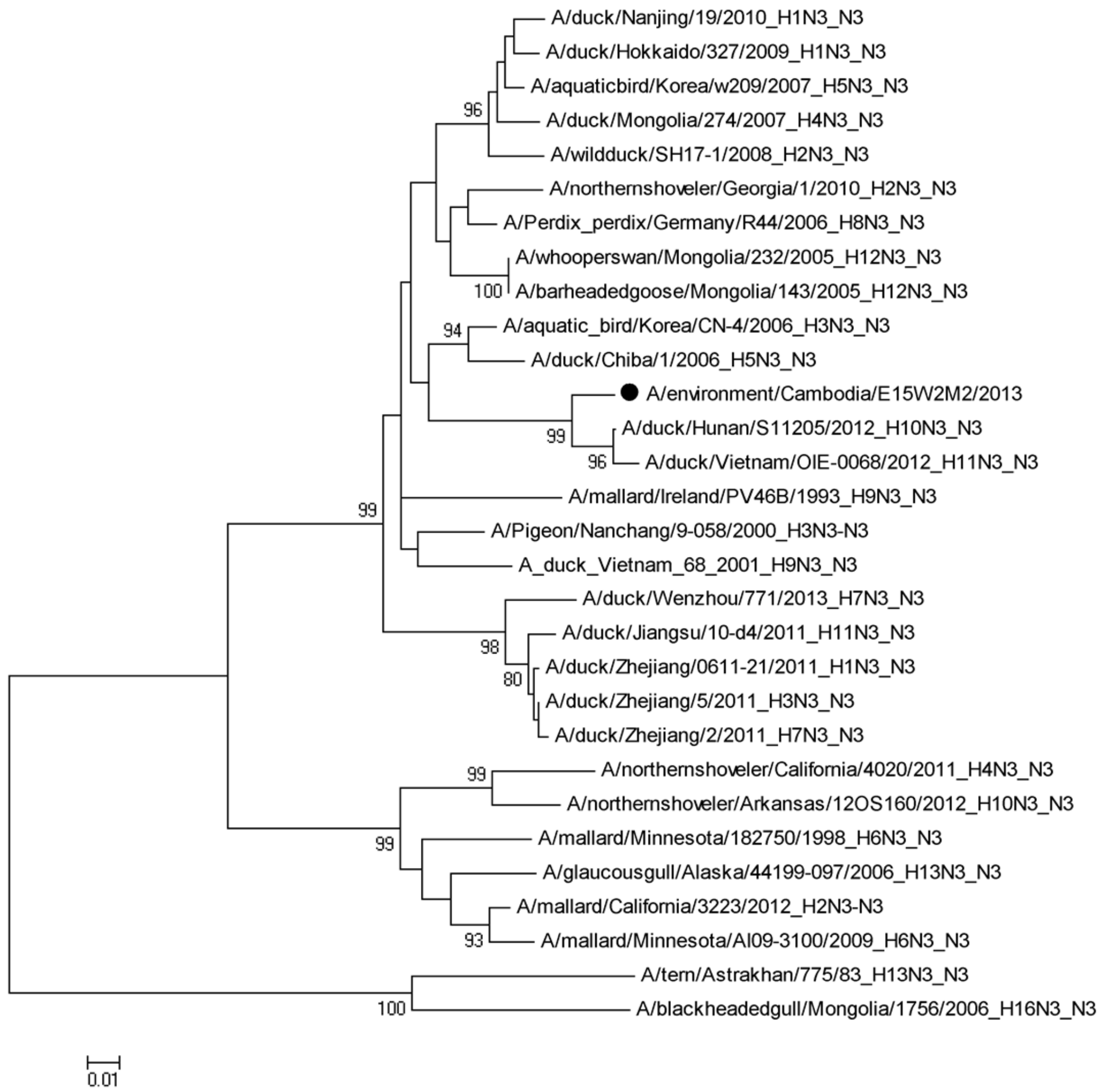

A/duck/Nanjing/19/2010_H1N3_N3
A/duck/Hokkaido/327/2009_H1N3_N3
A/aquaticbird/Korea/w209/2007_H5N3_N3
A/duck/Mongolia/274/2007_H4N3_N3
A/wildduck/SH17-1/2008_H2N3_N3
A/northernshoveler/Georgia/1/2010_H2N3_N3
A/Perdix_perdix/Germany/R44/2006_H8N3_N3
A/whooperswan/Mongolia/232/2005_H12N3_N3
A/barheadedgoose/Mongolia/143/2005_H12N3_N3
A/aquatic_bird/Korea/CN-4/2006_H3N3_N3
A/duck/Chiba/1/2006_H5N3_N3
A/environment/Cambodia/E15W2M2/2013
A/duck/Hunan/S11205/2012_H10N3_N3
A/duck/Vietnam/OIE-0068/2012_H11N3_N3
A/mallard/Ireland/PV46B/1993_H9N3_N3
A/Pigeon/Nanchang/9-058/2000_H3N3-N3
A_duck_Vietnam_68_2001_H9N3_N3
A/duck/Wenzhou/771/2013_H7N3_N3
A/duck/Jiangsu/10-d4/2011_H11N3_N3
A/duck/Zhejiang/0611-21/2011_H1N3_N3
A/duck/Zhejiang/5/2011_H3N3_N3
A/duck/Zhejiang/2/2011_H7N3_N3
A/northernshoveler/California/4020/2011_H4N3_N3
A/northernshoveler/Arkansas/12OS160/2012_H10N3_N3
A/mallard/Minnesota/182750/1998_H6N3_N3
A/glaucousgull/Alaska/44199-097/2006_H13N3_N3
A/mallard/California/3223/2012_H2N3-N3
A/mallard/Minnesota/AI09-3100/2009_H6N3_N3
A/tern/Astrakhan/775/83_H13N3_N3
A/blackheadedgull/Mongolia/1756/2006_H16N3_N3
96
100
94
99
96
99
98
80
99
99
93
100
0.01

N5

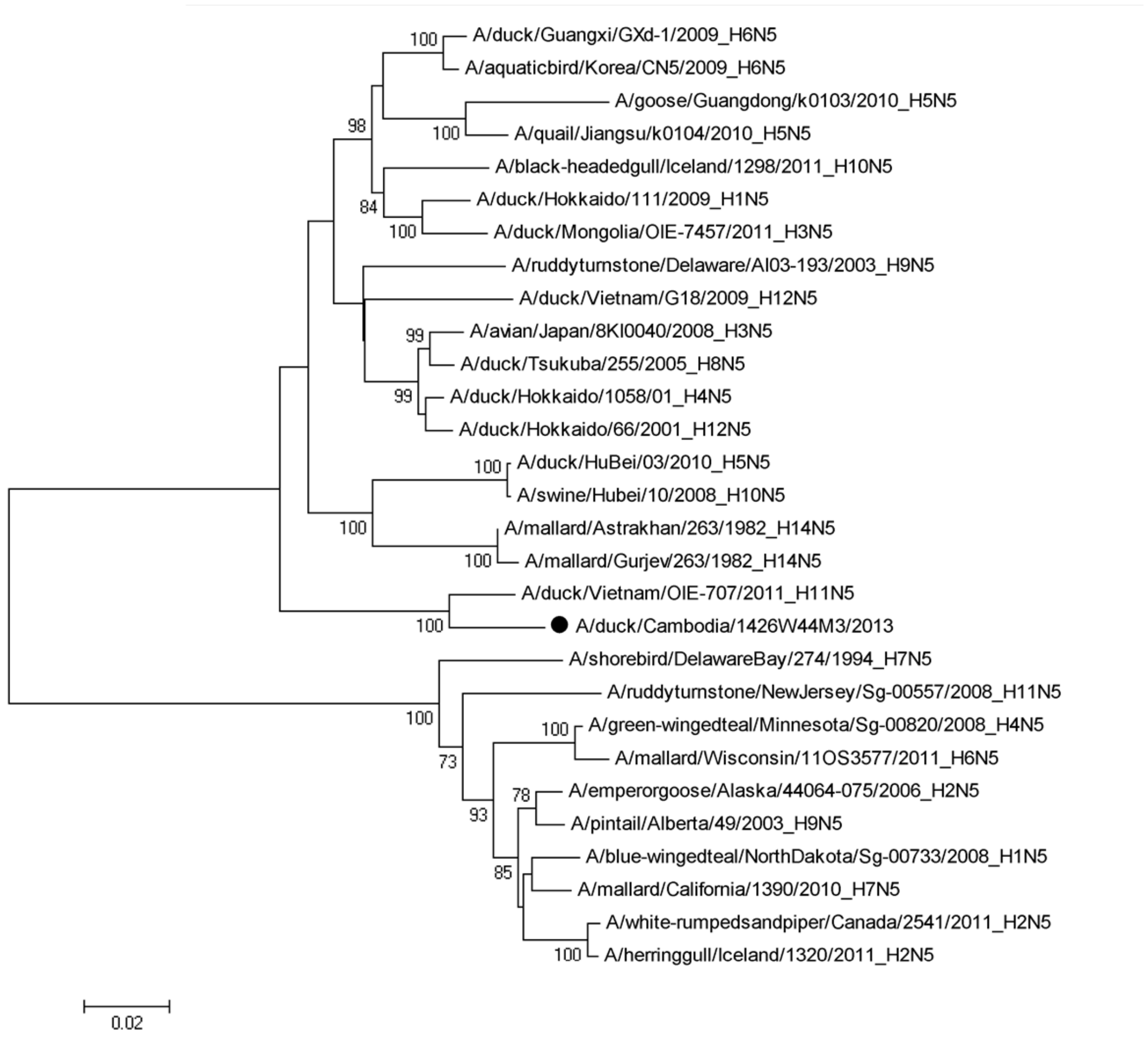
A/duck/Guangxi/GXd-1/2009_H6N5
A/aquaticbird/Korea/CN5/2009_H6N5
A/goose/Guangdong/k0103/2010_H5N5
A/quail/Jiangsu/k0104/2010_H5N5
A/black-headedgull/Iceland/1298/2011_H10N5
A/duck/Hokkaido/111/2009_H1N5
A/duck/Mongolia/OIE-7457/2011_H3N5
A/ruddyturnstone/Delaware/AI03-193/2003_H9N5
A/duck/Vietnam/G18/2009_H12N5
A/avian/Japan/8KI0040/2008_H3N5
A/duck/Tsukuba/255/2005_H8N5
A/duck/Hokkaido/1058/01_H4N5
A/duck/Hokkaido/66/2001_H12N5
A/duck/HuBei/03/2010_H5N5
A/swine/Hubei/10/2008_H10N5
A/mallard/Astrakhan/263/1982_H14N5
A/mallard/Gurjev/263/1982_H14N5
A/duck/Vietnam/OIE-707/2011_H11N5
A/duck/Cambodia/1426W44M3/2013
A/shorebird/DelawareBay/274/1994_H7N5
A/ruddyturnstone/NewJersey/Sg-00557/2008_H11N5
A/green-wingedteal/Minnesota/Sg-00820/2008_H4N5
A/mallard/Wisconsin/11OS3577/2011_H6N5
A/emperorgoose/Alaska/44064-075/2006_H2N5
A/pintail/Alberta/49/2003_H9N5
A/blue-wingedteal/NorthDakota/Sg-00733/2008_H1N5
A/mallard/California/1390/2010_H7N5
A/white-rumpedsandpiper/Canada/2541/2011_H2N5
A/herringgull/Iceland/1320/2011_H2N5
100
98
84
99
73
93
78
85
0.02

N6

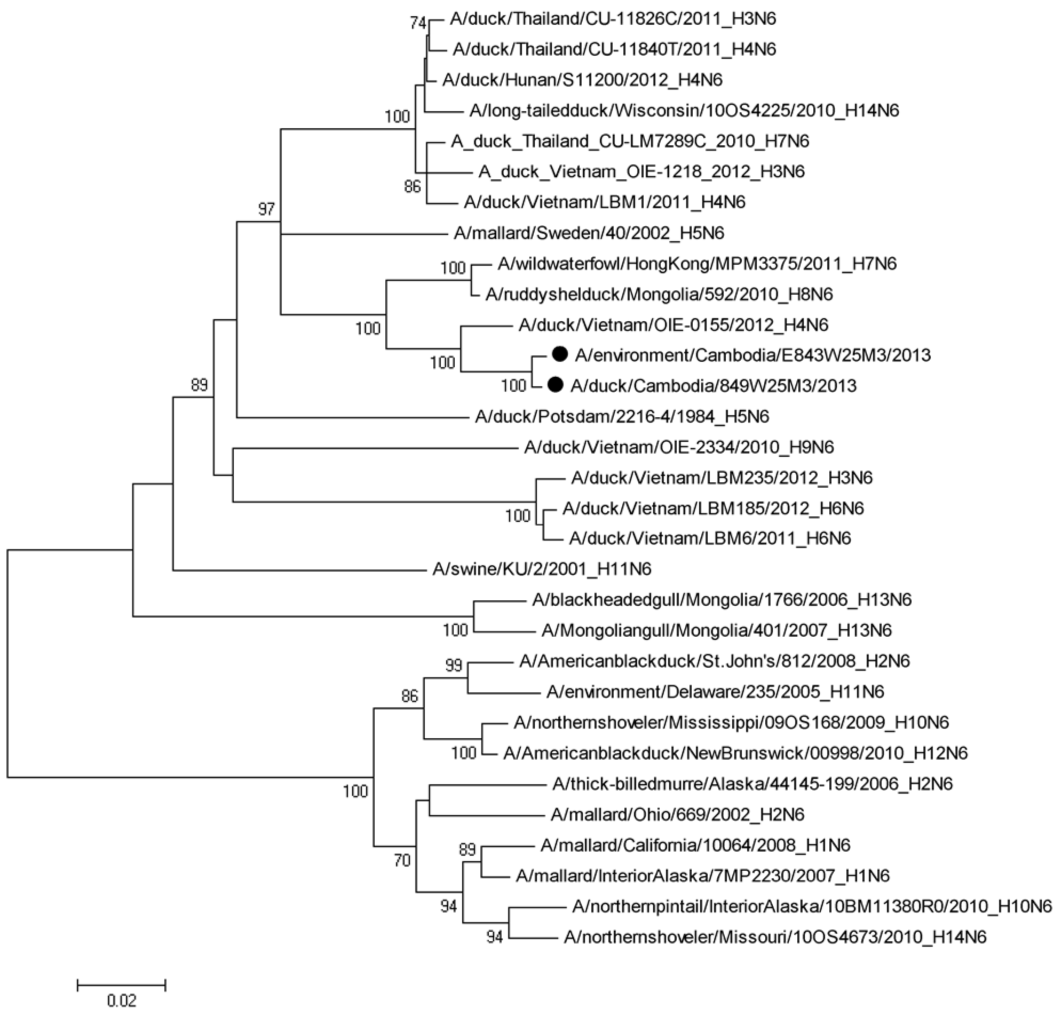
74
A/duck/Thailand/CU-11826C/2011_H3N6
A/duck/Thailand/CU-11840T/2011_H4N6
A/duck/Hunan/S11200/2012_H4N6
100
A/long-tailedduck/Wisconsin/10OS4225/2010_H14N6
A_duck_Thailand_CU-LM7289C_2010_H7N6
A_duck_Vietnam_OIE-1218_2012_H3N6
86
A/duck/Vietnam/LBM1/2011_H4N6
97
A/mallard/Sweden/40/2002_H5N6
100
A/wildwaterfowl/HongKong/MPM3375/2011_H7N6
A/ruddyshelduck/Mongolia/592/2010_H8N6
100
A/duck/Vietnam/OIE-0155/2012_H4N6
100
A/environment/Cambodia/E843W25M3/2013
100
A/duck/Cambodia/849W25M3/2013
89
A/duck/Potsdam/2216-4/1984_H5N6
A/duck/Vietnam/OIE-2334/2010_H9N6
A/duck/Vietnam/LBM235/2012_H3N6
100
A/duck/Vietnam/LBM185/2012_H6N6
A/duck/Vietnam/LBM6/2011_H6N6
A/swine/KU/2/2001_H11N6
A/blackheadedgull/Mongolia/1766/2006_H13N6
100
A/Mongoliangull/Mongolia/401/2007_H13N6
99
A/Americanblackduck/St.John's/812/2008_H2N6
86
A/environment/Delaware/235/2005_H11N6
A/northernshoveler/Mississippi/09OS168/2009_H10N6
100
A/Americanblackduck/NewBrunswick/00998/2010_H12N6
100
A/thick-billedmurre/Alaska/44145-199/2006_H2N6
A/mallard/Ohio/669/2002_H2N6
89
A/mallard/California/10064/2008_H1N6
70
A/mallard/InteriorAlaska/7MP2230/2007_H1N6
94
A/northernpintail/InteriorAlaska/10BM11380R0/2010_H10N6
94
A/northernshoveler/Missouri/10OS4673/2010_H14N6
0.02

N8

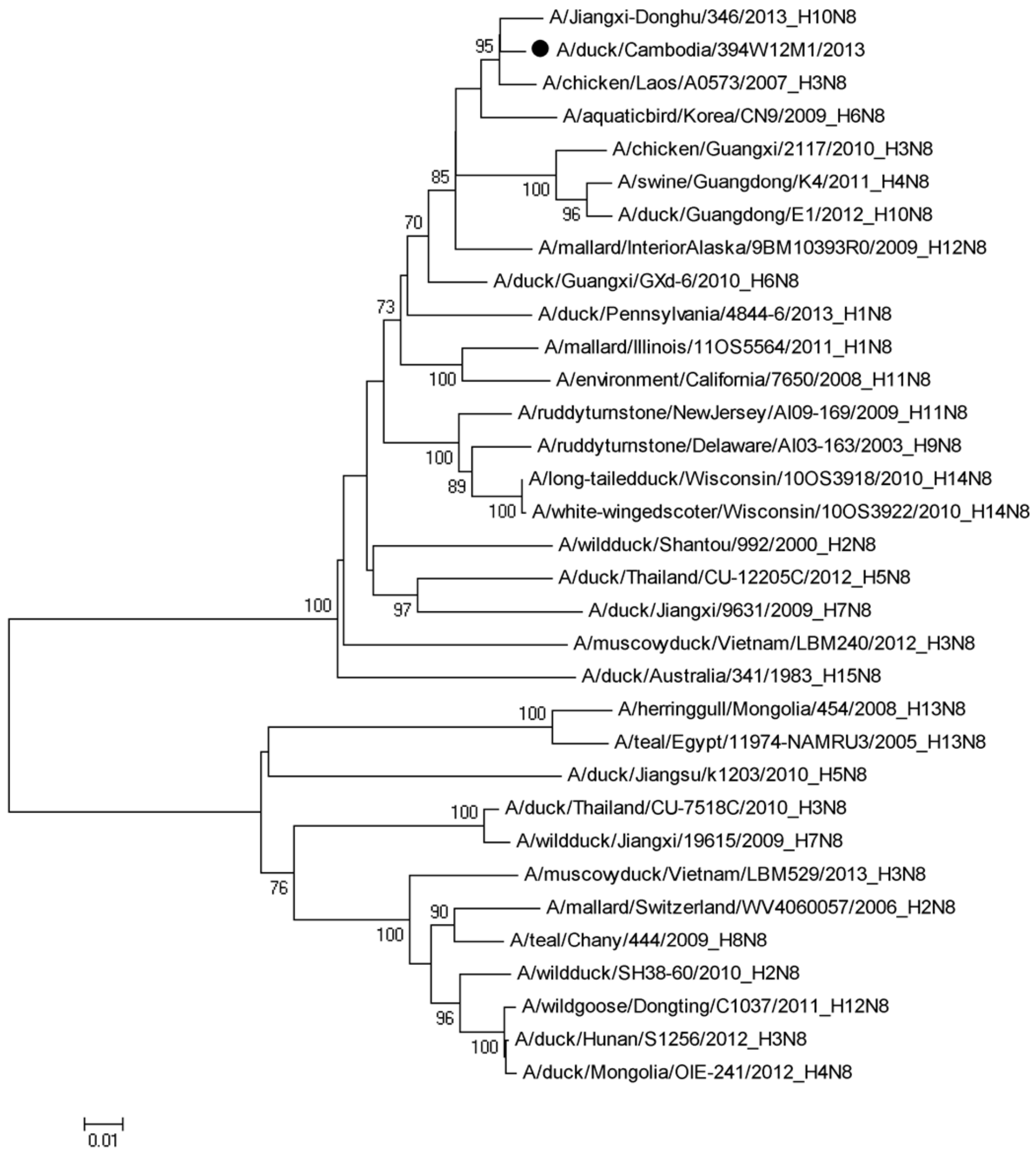
A/Jiangxi-Donghu/346/2013_H10N8
A/duck/Cambodia/394W12M1/2013
A/chicken/Laos/A0573/2007_H3N8
A/aquaticbird/Korea/CN9/2009_H6N8
A/chicken/Guangxi/2117/2010_H3N8
A/swine/Guangdong/K4/2011_H4N8
A/duck/Guangdong/E1/2012_H10N8
A/mallard/InteriorAlaska/9BM10393R0/2009_H12N8
A/duck/Guangxi/GXd-6/2010_H6N8
A/duck/Pennsylvania/4844-6/2013_H1N8
A/mallard/Illinois/11OS5564/2011_H1N8
A/environment/California/7650/2008_H11N8
A/ruddyturnstone/NewJersey/AI09-169/2009_H11N8
A/ruddyturnstone/Delaware/AI03-163/2003_H9N8
A/long-tailedduck/Wisconsin/10OS3918/2010_H14N8
A/white-wingedscoter/Wisconsin/10OS3922/2010_H14N8
A/wildduck/Shantou/992/2000_H2N8
A/duck/Thailand/CU-12205C/2012_H5N8
A/duck/Jiangxi/9631/2009_H7N8
A/muscovyduck/Vietnam/LBM240/2012_H3N8
A/duck/Australia/341/1983_H15N8
A/herringgull/Mongolia/454/2008_H13N8
A/teal/Egypt/11974-NAMRU3/2005_H13N8
A/duck/Jiangsu/k1203/2010_H5N8
A/duck/Thailand/CU-7518C/2010_H3N8
A/wildduck/Jiangxi/19615/2009_H7N8
A/muscovyduck/Vietnam/LBM529/2013_H3N8
A/mallard/Switzerland/WV4060057/2006_H2N8
A/teal/Chany/444/2009_H8N8
A/wildduck/SH38-60/2010_H2N8
A/wildgoose/Dongting/C1037/2011_H12N8
A/duck/Hunan/S1256/2012_H3N8
A/duck/Mongolia/OIE-241/2012_H4N8
95
85
100
96
70
73
100
100
89
100
97
100
100
100
76
100
90
96
100
0.01

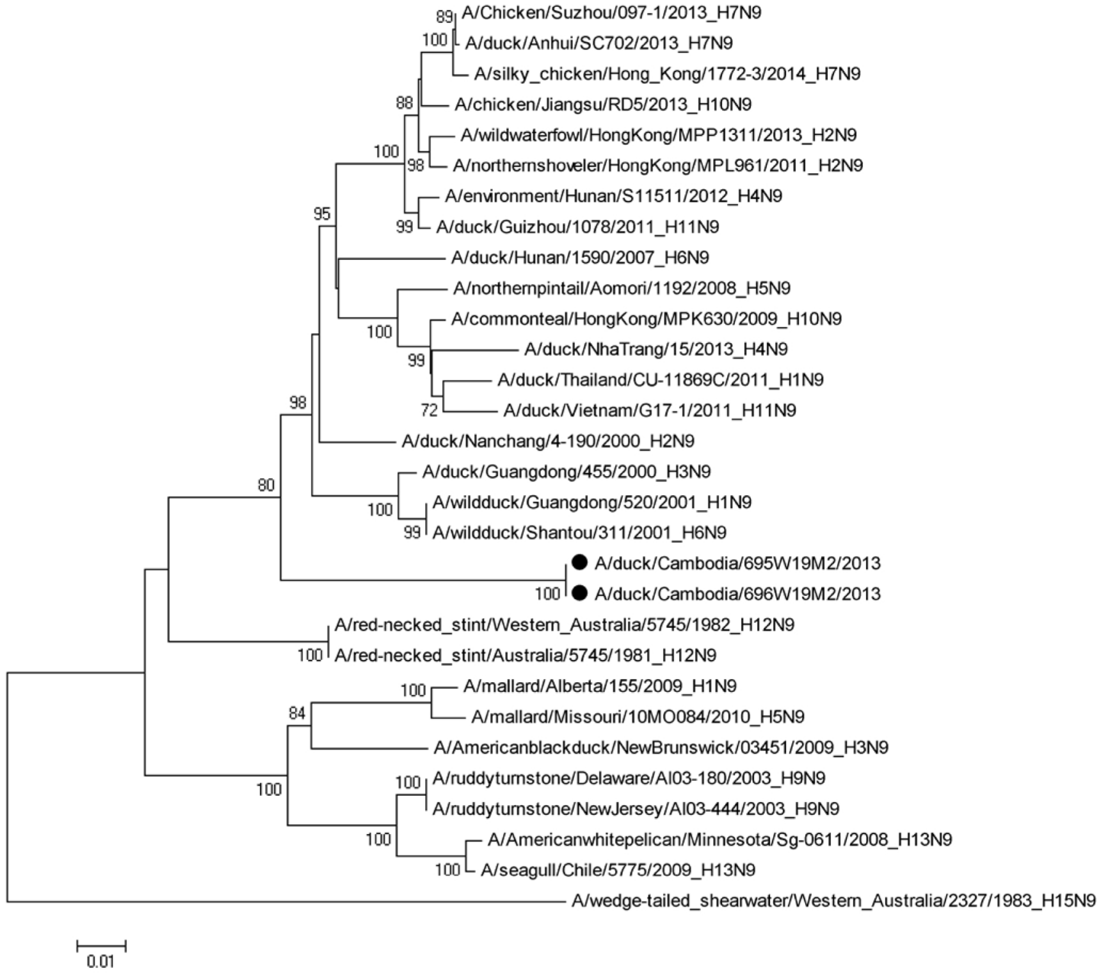

Supplementary Figure S3 Neighbor-joining phylogenetic trees of the neuraminidase (NA) genes of avian influenza viruses detected during live bird market surveillance in Cambodia. Viruses collected during the present market study are denoted by a black circle. The phylogenetic trees were constructed in MEGA5. Bootstraps greater than 70 generated from 1,000 replicates are shown at branch nodes. The scale bar represents the number of nucleotide substitutions per site.
