# Supplementary material for: Intense circulation of A/H5N1 and other avian influenza viruses in Cambodian live-bird markets with serological evidence of sub-clinical human infections
Source: Emerg Microbes Infect. 2016 Jul 20;5(7):e70–. doi: 10.1038/emi.2016.69 (PMC5141262; doi:10.1038/emi.2016.69)
Supplement: Supplementary Table S1 [file emi201669x4.pdf]

Supplementary Table 1. GenBank/GISAID accession numbers for all Cambodian strains included in the phylogenetic analyses

Strain name	PB2	PB1	PA	HA	NP	NA	M	NS	Sequences generated in the present study
**A/duck/Cambodia/46W2M4/2013**	**KF001423**	**KF001424**	**KF001425**	**KF001426**	**KF001427**	**KF001428**	**KF001429**	**KF001430**	Yes
**A/duck/Cambodia/143W4M4/2013**	**KF369233**	**KF001433**	**KF369234**	**KF001434**	**KF001435**	**KF001436**	**KF001437**	**KF001438**	Yes
**A/duck/Cambodia/191W5M4/2013**	**KF001439**	**KF001440**	**KF001441**	**KF001442**	**KF001443**	**KF001444**	**KF001445**	**KF001446**	Yes
**A/duck/Cambodia/202W6M1/2013**	**KF369235**	**KF001447**	**KF369236**	**KF001448**	**KF001449**	**KF001450**	**KF001451**	**KF001452**	Yes
**A/duck/Cambodia/250W7M1/2013**	**KF001453**	**KF369237**	**KF001454**	**KF001455**	**KF001456**	**KF001457**	**KF001458**	**KF001459**	Yes
**A/duck/Cambodia/261W7M2/2013**	**KF369238**	**KF369239**	**KF001460**	**KF001461**	**KF001462**	**KF001463**	**KF001464**	**KF001465**	Yes
A/duck/Cambodia/11W2M1/2013	N/A	N/A	N/A	KF001419	N/A	KF001420	N/A	N/A	Yes
A/duck/Cambodia/33W2M3/2013	N/A	N/A	N/A	KF001421	N/A	KF001422	N/A	N/A	Yes
A/duck/Cambodia/587W16M1/2013	**EPI587505**	**EPI587506**	EPI587507	**EPI587508**	**EPI587509**	**EPI587510**	**EPI587511**	**EPI587512**	Yes
A/environment/Cambodia/E772W23M1/2013	N/A	**EPI587513**	N/A	**EPI587514**	**EPI587515**	**EPI587516**	**EPI587517**	**EPI587518**	Yes
A/environment/Cambodia/C823W25M1/2013	EPI588984	EPI588985	EPI588986	EPI588987	**EPI588988**	**EPI588989**	**EPI588990**	**EPI588991**	Yes
A/environment/Cambodia/E842W25M3/2013	EPI588993	EPI588994	EPI588995	**EPI588996**	EPI588997	**EPI588998**	**EPI588999**	**EPI589000**	Yes
A/environment/Cambodia/E846W25M3/2013	EPI589001	EPI589002	EPI589003	**EPI589004**	EPI589005	N/A	**EPI589006**	**EPI589007**	Yes
A/environment/Cambodia/E866W27M1/2013	**EPI589008**	EPI589009	**EPI589010**	**EPI589011**	**EPI589012**	**EPI589014**	**EPI589015**	**EPI589016**	Yes
A/environment/Cambodia/E878W27M2/2013	**EPI589017**	EPI589018	**EPI589019**	**EPI589020**	**EPI589021**	EPI589022	**EPI589023**	**EPI589024**	Yes
A/environment/Cambodia/E938W29M3/2013	EPI589105	EPI589106	**EPI589107**	**EPI589108**	**EPI589109**	**EPI589110**	**EPI589111**	**EPI589112**	Yes
A/environment/Cambodia/E990W31M3/2013	EPI589114	N/A	EPI589115	**EPI589116**	EPI589117	**EPI589118**	**EPI589120**	**EPI589121**	Yes
A/environment/Cambodia/E1038W33M3/2013	EPI589122	EPI589123	**EPI589124**	**EPI589125**	**EPI589126**	**EPI589127**	**EPI589128**	**EPI589129**	Yes
A/environment/Cambodia/E1082W35M3/2013	EPI589339	N/A	**EPI587499**	EPI587500	**EPI497944**	**EPI537678**	**EPI587501**	**EPI537679**	Yes
A/environment/Cambodia/E1106W37M1/2013	EPI589130	EPI589131	**EPI589132**	EPI589133	**EPI589134**	**EPI589135**	**EPI589136**	**EPI589137**	Yes
A/environment/Cambodia/E1144W37M4/2013	EPI589138	N/A	EPI589139	**EPI589140**	**EPI589141**	**EPI589142**	**EPI589143**	EPI589144	Yes
A/environment/Cambodia/C1154W38M1/2013	N/A	N/A	N/A	**KF918519**	**KF918520**	N/A	N/A	N/A	Yes
A/duck/Cambodia/1281W40M3/2013	**EPI587502**	EPI587503	**EPI587504**	**EPI497952**	**EPI497953**	**EPI497954**	**EPI497955**	**EPI497956**	Yes
A/duck/Cambodia/1295W40M4/2013	N/A	N/A	N/A	**EPI497957**	**EPI497958**	N/A	N/A	N/A	Yes
A/environment/Cambodia/E1396W44M1/2013	**EPI589176**	EPI589177	**EPI589178**	**EPI589179**	**EPI589180**	**EPI589181**	**EPI589182**	**EPI589183**	Yes
A/Cambodia/X0123311/2013	**KF001369**	**KF001370**	**KF001371**	**KF001372**	**KF001373**	**KF001374**	**KF001375**	**KF001376**	No
A/Cambodia/X0123312/2013	KF001377	**KF001378**	**KF001379**	**KF001380**	**KF001381**	**KF001382**	**KF001383**	**KF001384**	No
A/chicken/Cambodia/X0124310/2013	KF001483	**KF001484**	**KF001485**	**KF001486**	**KF001487**	**KF001488**	**KF001489**	**KF001490**	No
A/Cambodia/X0125302/2013	KF001385	**KF001386**	**KF001387**	**KF001388**	**KF001389**	**KF001390**	**KF001391**	**KF001392**	No
A/Cambodia/X0207301/2013	**KF001393**	**KF001394**	**KF001395**	**KF001396**	**KF001397**	**KF001398**	**KF001399**	**KF001400**	No
A/Cambodia/X0212301/2013	KF001401	**KF001402**	**KF001403**	**KF001404**	**KF001405**	**KF001406**	**KF001407**	**KF001408**	No
A/Cambodia/X0219301/2013	**KF001409**	**KF001410**	**KF001411**	**KF001412**	**KF001413**	**KF001414**	**KF001415**	**KF001416**	No
A/duck/Cambodia/X0220302/2013	N/A	N/A	N/A	**KF001497**	N/A	**KF001498**	N/A	N/A	No
A/Chicken/Cambodia/X0305302/2013	**KF001499**	**KF001500**	**KF001501**	**KF001502**	**KF001503**	**KF001504**	**KF001505**	**KF001506**	No
A/Cambodia/X0628313/2013	**KF918453**	**KF918454**	**KF918455**	**KF918456**	**KF918457**	**KF918458**	**KF918459**	**KF918460**	No
A/Cambodia/X0808305/2013	**KF918461**	**KF918462**	**KF918463**	**KF918464**	**KF918465**	**KF918466**	**KF918467**	**KF918468**	No
A/Cambodia/X0810301/2013	**EPI537652**	**EPI537653**	**KF918469**	**KF918470**	**KF918471**	**KF918472**	**KF918473**	**KF918474**	No
A/chicken/Cambodia/X0815301/2013	**KF918475**	**KF918476**	**KF918477**	**KF918478**	**KF918479**	**KF918480**	**KF918481**	**KF918482**	No
A/Cambodia/X0817302/2013	**KF918487**	**KF918488**	**KF918489**	**KF918490**	**KF918491**	**KF918492**	**KF918493**	**KF918494**	No
A/Cambodia/X0828324/2013	**KF918495**	**KF918496**	**KF918497**	**KF918498**	**KF918499**	**KF918500**	**KF918501**	**KF918502**	No
A/Cambodia/X0913301/2013	**KF918503**	**KF918504**	**KF918505**	**KF918506**	**KF918507**	**KF918508**	**KF918509**	**KF918510**	No
A/Cambodia/X0916322/2013	**KF918511**	**KF918512**	**KF918513**	**KF918514**	**KF918515**	**KF918516**	**KF918517**	**KF918518**	No
A/Cambodia/X1024307/2013	**KF918527**	**KF918528**	**KF918529**	**KF918530**	**KF918531**	**KF918532**	**KF918533**	**KF918534**	No
A/Cambodia/X1030304/2013	**EPI537641**	**EPI537642**	**EPI537643**	**EPI497961**	**EPI537644**	**EPI497962**	**EPI537645**	**EPI537646**	No
A/Cambodia/X1107305/2013	N/A	N/A	N/A	EPI537657	N/A	EPI537658	N/A	N/A	No
A/duck/Cambodia/W0530392/2012	N/A	N/A	N/A	**KF369231**	N/A	KF369232	N/A	N/A	No

Strain name	PB2	PB1	PA	HA	NP	NA	M	NS	Sequences generated in the present study
A/Cambodia/W0526301/2012	**KF369211**	**KF369212**	**KF369213**	**KF369214**	**KF369215**	**KF369216**	**KF369217**	**KF369218**	No
A/chicken/Cambodia/W0530389/2012	**KF369219**	**KF369220**	**KF369221**	KF369222	**KF369223**	**KF369224**	**KF369225**	**KF369226**	No
A/chicken/Cambodia/089LC1/2011	N/A	N/A	N/A	**JQ714240**	N/A	N/A	N/A	N/A	No
A/Cambodia/V0719348/2011	N/A	N/A	N/A	**JQ714217**	N/A	N/A	JQ714218	JQ714219	No
A/environment/Cambodia/C425D/2011	**JQ673591**	**JQ673595**	**JQ673599**	**JQ673603**	**JQ673610**	**JQ673614**	**JQ673618**	**JQ673622**	No
A/Cambodia/V0606311/2011	**JN588929**	**JQ689200**	**JN588899**	JN588809	JN588866	**JN588860**	**JN588830**	**JN588882**	No
A/chicken/Cambodia/008LC1/2011	**JN588937**	**JN588922**	**JN588908**	**JN588824**	**JN588876**	**JN588854**	**JN588839**	**JN588892**	No
A/Cambodia/V0219301/2011	**JN588926**	**JN588911**	**JN588896**	**JN588806**	JN588863	**JN588857**	**JN588827**	**JN588879**	No
A/Cambodia/V0813302/2011	N/A	N/A	N/A	JQ714220	**JQ714221**	N/A	JQ714222	**JQ714223**	No
A/duck/Cambodia/072D1/2011	JQ701717	JQ701718	**JQ714251**	JQ701719	**JQ701720**	JQ701721	**JQ701722**	**JQ701723**	No
A/duck/Cambodia/072D6/2011	**JQ714210**	**JQ714211**	**KF369202**	JQ714212	JQ714213	**JQ714214**	JQ714215	**JQ714216**	No
A/duck/Cambodia/PV027D1/2010	**JN588935**	**JN588920**	**JN588906**	**JN588821**	**JN588874**	**JN588851**	**JN588949**	**JN588890**	No
A/chicken/Cambodia/TKCMB5T/2010	**JN588932**	**JN588917**	**JN588903**	JN588815	**JN588871**	JN588845	**JN588836**	JN588887	No
A/Cambodia/U0417030/2010	**JN588924**	**JN588909**	**JN588894**	**JN588804**	**JN588861**	**JN588855**	**JN588825**	**JN588877**	No
A/chicken/Cambodia/TLC1/2009	**JN588930**	**JN588914**	**JN588900**	**JN588811**	**JN588868**	**JN588841**	**JN588832**	**JN588884**	No
A/chicken/Cambodia/67F1/2008	N/A	N/A	**JN588943**	JN588938	JN588941	JN588942	JN588939	JN588940	No
A/chicken/Cambodia/67F8/2008	N/A	N/A	N/A	**JN588944**	**JN588947**	**JN588948**	**JN588945**	**JN588946**	No
A/Cambodia/S1211394/2008	**JN588923**	**HQ200601**	**JN588893**	**HQ200596**	**HQ200599**	**HQ200598**	**HQ200597**	**HQ200600**	No
A/chicken/Cambodia/LC1AL/2007	**HQ200577**	**HQ200578**	**HQ200579**	**HQ200575**	**HQ200576**	**HQ200574**	**HQ200580**	**HQ200581**	No
A/chicken/Cambodia/LC3AL/2007	**HQ200585**	HQ200586	HQ200587	HQ200583	HQ200584	HQ200582	HQ200588	HQ200589	No
A/Cambodia/R0405050/2007	HQ200569	**HQ200570**	**HQ200571**	**FJ225472**	**HQ200568**	**FJ225473**	**HQ200572**	**HQ200573**	No
A/Cambodia/Q0321176/2006	**HQ200422**	N/A	**EPI588983**	**HQ200417**	HQ200420	**HQ200418**	**HQ200419**	**HQ200421**	No
A/Cambodia/Q0405047/2006	N/A	N/A	N/A	HQ200423	**HQ200426**	**HQ200424**	**HQ200425**	**HQ200427**	No
A/chicken/Cambodia/LC/2006	HQ200449	**HQ200448**	HQ200447	HQ200442	HQ200445	HQ200443	HQ200444	**HQ200446**	No
A/duck/Cambodia/D14AL/2006	**HQ200457**	**HQ200456**	**HQ200455**	**HQ200450**	**HQ200453**	**HQ200451**	**HQ200452**	**HQ200454**	No
A/duck/Cambodia/D3PV/2006	**HQ200479**	**HQ200478**	**HQ200480**	**HQ200475**	**HQ200481**	**HQ200476**	**HQ200477**	**HQ200482**	No
A/Cambodia/JP52a/2005	N/A	N/A	N/A	**EF456805**	N/A	**EF456793**	N/A	N/A	No
A/Cambodia/P0322095/2005	**HQ200462**	**HQ200463**	**HQ200464**	**HQ200458**	**HQ200465**	**HQ200459**	**HQ200461**	**HQ200460**	No
A/chicken/Cambodia/022LC2b/2005	**HQ200530**	HQ200534	**HQ200532**	**HG664945**	**HQ200531**	**EF473077**	HQ200533	**HQ200529**	No
A/Cambodia/408008/2005	**HQ664943**	**HQ664942**	**HQ664941**	HQ664938	HQ664940	**HQ664939**	HQ664944	**HQ664945**	No
A/goose/Cambodia/28/2004	N/A	N/A	N/A	**EF473070**	N/A	**EF473071**	**EF473072**	N/A	No

Accession numbers for strains with complete gene sequences available are shown in bold type
Accession numbers for strains with partial gene sequences available are shown in normal type
GenBank accession numbers are shown in black type; GISAID accession numbers are shown in blue type

Supplementary Table 1 GenBank/GISAID accession numbers for all Cambodian strains included in the phylogenetic analyses
